# Ion-Sieving Dual-Scale Asymmetric Cellulose Membrane as a Sustainable Paper-Based Separator for Ultra-Stable Zinc Anodes

**DOI:** 10.1007/s40820-026-02165-0

**Published:** 2026-03-27

**Authors:** Xinlong Liu, Junze Zhang, Cuiqin Fang, Yana Xiao, Yujue Yang, Shuai Wang, Qingjun Yang, Yaopeng Wu, Bingang Xu

**Affiliations:** 1 Research Institute for Intelligent Wearable Systems, The Hong Kong Polytechnic University, Kowloon, 999077 Hong Kong, People’s Republic of China; 2 Research Centre for Resources Engineering Towards Carbon Neutrality, The Hong Kong Polytechnic University, Kowloon, 999077 Hong Kong, People’s Republic of China

**Keywords:** Cellulose separator, Glass fiber, Dual-scale, Biodegradable, Zinc anode

## Abstract

Conventional glass fiber separators used in aqueous zinc-ion batteries (ZIBs) are inadequate in suppressing Zn dendrite growth and parasitic reactions due to unregulated ion transport. Here, we design a fully biodegradable and dual-scale asymmetric paper-based membrane that synergistically couples a macroporous paper scaffold with a surface layer of carboxylated nanoporous cellulose nanofibers (CNFs) for ion regulation. This dual-scale architecture establishes coordination-assisted ion-hopping pathways via Zn^2+^–COOH interactions, homogenizing Zn^2+^ flux to enable uniform nucleation and inhibit dendrites. Simultaneously, the nanoporous and negatively charged CNF layer functions as an ion sieve, preferentially conducting Zn^2+^ while restricting water mobility and polyiodide shuttling, thereby mitigating side reactions. When deployed as a separator, the membrane enables an ultra-stable Zn||Zn symmetric cell cycling over 1,900 h at 1.0 mA cm^−2^ and an average Coulombic efficiency of 97.3% in Zn||Cu cells, achieving a sixfold lifespan extension over commercial glass fiber separators. The corresponding Zn||I_2_ full cell retains a specific capacity of 172.8 mAh g^−1^ after 4,000 cycles at 2.0 A g^−1^, underscoring its efficacy in suppressing shuttle effects. This cellulose-based design reduces separator cost by 83% while ensuring full biodegradability, offering a practical and sustainable pathway toward high-performance ZIBs.

**Supplementary Information:**

The online version contains supplementary material available at 10.1007/s40820-026-02165-0.

## Highlights

- A cost-effective and fully biodegradable dual-scale paper-based separator is engineered by intertwining macroporous rice paper with mesoporous nanofibers.
- Nanoporous ion sieving and molecular confined pathways effectively suppress polyiodide shuttling and water-induced side reactions.
- Regulated ion transport induces a uniform solid-electrolyte interphase, enabling dendrite-free anodes and high Coulombic efficiency.

**Supplementary Information:**

The online version contains supplementary material available at 10.1007/s40820-026-02165-0.

## Graphical abstract

**Figure:**
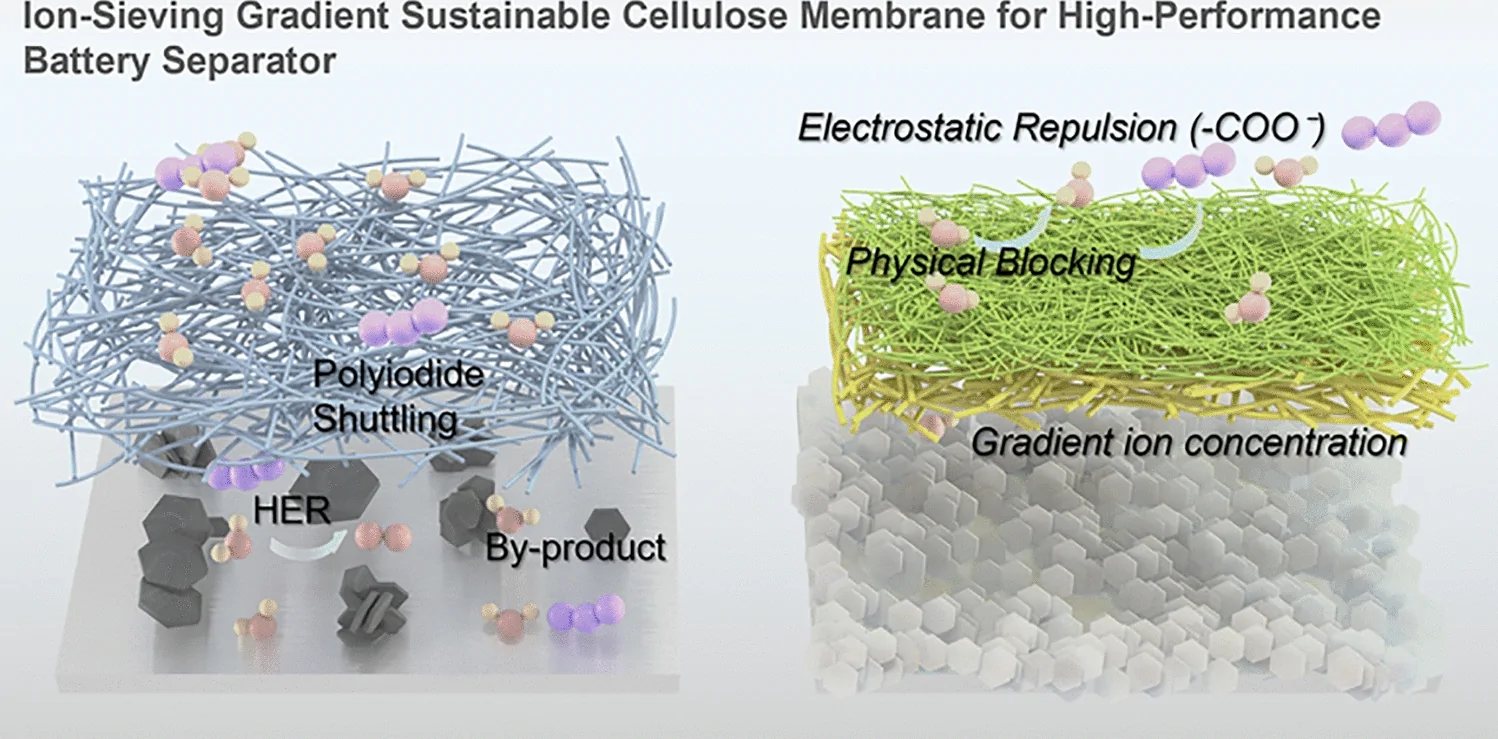

## Introduction

Aqueous zinc-ion batteries (ZIBs) hold significant interest as next-generation energy storage devices by intrinsic virtues of Zn anode including high safety, environmental inertness, natural abundance, and relatively high theoretical capacity (760 mAh g^−1^ or 5,855 mAh cm^−3^) [1]. However, the commercialization of ZIBs is substantially hampered by practical issues at the anode–electrolyte interface. These include parasitic side reactions, which lead to the formation of inert by-products like zinc hydroxide sulfate (ZHS) and cause low Coulombic efficiency (CE), as well as the uncontrollable growth of zinc dendrites during repeated plating/stripping cycles, which compromises battery safety and longevity [2]. Addressing these challenges is crucial to unlock the full potential of aqueous zinc-ion batteries for practical utilization [3].

Substantial efforts have been directed toward alleviating these challenges through strategies such as Zn substrate modification [4, 5], electrolyte engineering [6–9], interface reconstruction [10, 11], and separator design [12, 13], achieving notable effects on high-performance anodes. Among these, separator engineering stands out as a particularly versatile and effective approach to reinforce zinc anode performance [12, 13]. An ideal separator should not only serve as a physical barrier but also function as a regulator for ion transport and improved interfacial chemistry [14], which allows rational design on separator to enhance stability of Zn electrode. While commercial glass fiber (GF) separators offer high electrolyte uptake and ionic conductivity, their inherent limitations such as inadequate mechanical strength and an irregular pore structure often fail to uniformly distribute ion flux, thereby exacerbating dendrite growth [15]. Consequently, various GF hybrid separators have been explored to improve the lifetime of Zn anode. For instance, MOF-decorated GFs (e.g., UiO-66) functionalized GF with polar functional groups (with different functional groups –COOH [16, 17], –SO_3_H [18], –NH_2_ [19]) demonstrate ionic selectivity, enhancing the stability of both Zn||Zn and Zn||I₂ cells. Similar ion-sieving effects have been achieved using inorganic nanomaterials like halloysite nanotubes [20], silica nanosheet [21], and Fe nanoparticles [22]. Alternatively, electrospun fibrous membranes such as PAN [23–25], PVA/PAA [26], and PVA/HNTs [27] offer tunable porosity, controllable thickness, and facile functionalization, realizing dendrite-free deposition. Despite their promising performance, the high cost and complex fabrication processes associated with MOF-modified and electrospun separators present significant barriers to their large-scale, cost-effective application.

In pursuit of sustainable battery components, cellulose-based separators have recently emerged as attractive candidates for ZIBs, capitalizing on their eco-friendliness, biodegradability, and low cost [28, 29]. Cellulose materials, derived from abundant biomass, are characterized by rich polar functional groups, high mechanical strength, and inherent hydrophilicity, which are beneficial for electrolyte wettability and ion conduction, thereby rendering a dendrite-suppressing feature [15, 30–36]. Cellulose nanofibers (CNFs), particularly with their high aspect ratio and modifiable surface chemistry (e.g., carboxylation), can be engineered into multifunctional membranes. Especially, the polar groups (e.g., –COOH) on CNFs can provide numerous nucleation sites, modulate ion concentration gradients, and sequester free water molecules. However, the hydrogen bonding caused by oxygen-containing groups between interchains could lead to high crystallinity of cellulose, which blocks the diffusion pathway for Zn^2+^ especially at high rate, induces large polarization, and consequently causes dendrite formation [30]. This issue is compounded in water-saturated, macroporous cellulose structures, which can facilitate hydrogen evolution reactions (HER) and the accumulation of by-products over extended cycling.

Therefore, the development of a separator that concurrently ensures efficient Zn^2+^ flux regulation, robust interfacial stabilization, ultra-low cost, and complete biodegradability within a scalable architecture remains a critical challenge. Herein, we propose an innovative ion-sieving and dual-scale asymmetric cellulose architecture realized through a low-cost paper-based membrane. This design features a macroporous rice paper (RP) substrate that provides mechanical integrity and facilitates bulk ion transport, seamlessly integrated with a mesoporous layer of carboxyl-functionalized cellulose nanofibers (CNFs). This dual-scale architecture yields two synergistic functions: (1) Molecular sieving effect: The tailored nanopores and negatively charged surfaces within the CNF layer selectively facilitate the transport of Zn^2+^ ions while effectively blocking anions (e.g., SO_4_^2−^, polyiodides) and restricting water molecule mobility, thus mitigating parasitic reactions, corrosion, and shuttle effects. (2) Interfacial ion-flux homogenization: The abundant –COOH groups on the CNFs coordinate with Zn^2+^ ions, creating a guided ion diffusion pathway that homogenizes the Zn^2+^ flux at the electrode interface, fundamentally suppressing dendrite initiation and promoting planar zinc deposition. When utilized in separator in ZIBs, it demonstrates exceptional electrochemical performances among all Zn||Zn symmetric cell, Zn||SS half-cell, and Zn||I_2_ full cell, achieving 1,900 h at 1.0 mA cm^−2^, over 1250 cycles (average CE of ~ 97.3%), and ultra-long cycling stability (172.8 mAh g^−1^ at 2.0 A g^−1^ over 4,000 cycles), respectively. Remarkably, the all-cellulose membrane exhibits robust mechanical strength (exceeding 6 MPa) while retaining full biodegradability. This work underscores the immense potential of rationally designed paper-based separators in developing high-performance, sustainable, and economically viable zinc-ion batteries.

## Experimental Section

### Preparation of Cellulose Paper-Based Separator

Cellulose nanofibers (CNFs, content of carboxy groups with ~ 1.2 mmol g^−1^, length of fiber with 5.0–10 μm) were purchased from Tianjin Mujinjin Biotechnology Co., Ltd, which is synthesized using the TEMPO-oxidation method, a selective oxidation process that converts the primary hydroxyl groups at the C6 position of cellulose to carboxylate groups. The 3.0 g CNFs slurry was mixed with 1.0 mL deionized water to form a uniform suspension. After that, the mixture was pasted onto the rice paper and then dried in oven at 60 °C for 6 h to yield the dual-scale asymmetric cellulose separator (RP/CNF). The half rice paper and GF combining with CNF (HRP/CNF, GF/CNF) were also prepared in a similar method.

### Preparation of I_2_ and PANI Cathodes

The iodine (I₂) cathode was fabricated via a thermal infusion method. Commercial iodine crystals (Macklin, ≥ 99.8%) were directly blended with Ketjen black (KB) at a mass ratio of 6:4 (I_2_:KB) and manually homogenized for 10 min. The mixture was vacuum-sealed in a quartz tube and thermally treated at 80 °C for 4 h to obtain a KB-I₂ composite. For electrode preparation, the composite was combined with KB conductive additive and polyvinylidene fluoride (PVDF) binder at an 8:1:1 ratio (w/w), dispersed in N-methyl-2-pyrrolidone (NMP) to form a slurry, and uniformly coated onto stainless steel (SS) current collectors. After drying at 60 °C for 12 h, the electrode has an active material loading of 2.0–3.0 mg cm^−2^ for Zn–I_2_ cell assembly. The PANI cathode was prepared by mixing PANI:KB:PVDF in a ratio of 8:1:1 (w/w), then pasted onto the SS, and finally dried at the same condition.

### Radar Chart Processing Methodology

The dataset was normalized row-wise using min−max scaling to preserve the relative differences within each observation. For rows representing cost and thickness, an inverse transformation was applied to ensure lower raw values correspond to higher normalized scores (i.e., *x*_norm_ = 1−(*x−*min)/(max−min)). All normalized values were scaled to [0, 1], enabling direct visual comparison across dimensions in radar plots.

## Results and Discussion

### Mechanical and Electrochemical Advantages of Asymmetric Paper Membranes

The –COOH terminated CNFs were prepared using the TEMPO-catalyzed oxidization method. This process, followed by ultrasonication treatment, yielded cellulose nanofibers with high surface charge, water dispersibility, and stability, suitable for diverse applications [37]. The SEM images of the as-synthesized CNFs are illustrated in Fig. S1, which shows the interconnected fiber framework with diameters of less than 10–20 nm and length of 5.0–10.0 μm. The highly –COOH terminated CNFs can produce a thin film with strong tensile strength via hydrogen bonding, with which it buffers the ion concentration at the interface to limit the dendrite formation. To our best knowledge, CNFs are only utilized as the main substrate material when preparing the membrane for aqueous rechargeable batteries, which is obtrusive to the commercialization due to the high price of CNFs. Therefore, we take the opposite approach to use CNFs as the secondary materials while applying the inexpensive rice paper as the primary, with the aim of emphasizing functionality and carrier properties, respectively. Compared with CNFs, the cheap rice paper and glass fiber from crude fibers are composed of microfibers with a diameter of up to 50 μm micrometers and length up to millimeters (Fig. S2), creating abundant large holes and interstices. This forms a perfect combination of CNFs and rice paper for the multifunctional and robust battery separator, in which the nanofibers fill the gaps within microfibers (Fig. S3a–i). As revealed by the cross-sectional SEM images, the CNF-loaded RP membrane exhibits a total thickness of approximately ~ 216 μm (Fig. S3j). Crucially, the magnified view (Fig. S3k) demonstrates that during preparation process, the nanoscale CNFs do not merely accumulate on the surface of the macroporous rice paper. Instead, driven by the pressure differential, the CNF deeply infiltrates and penetrates into the upper porous framework of the substrate. This distinct infiltration results in the formation of a “intertwined layer” bridging the dense upper CNF layer and the porous lower paper layer. Consequently, this architecture confirms the creation of a significant interpenetrated transition zone rather than a sharp interface, validating the structural continuity of the asymmetric membrane. In addition, the thin upper layer CNF shows that the integration of CNF barely contributes to the overall thickness of the membranes (Fig. S3l).

**Fig. 1:**
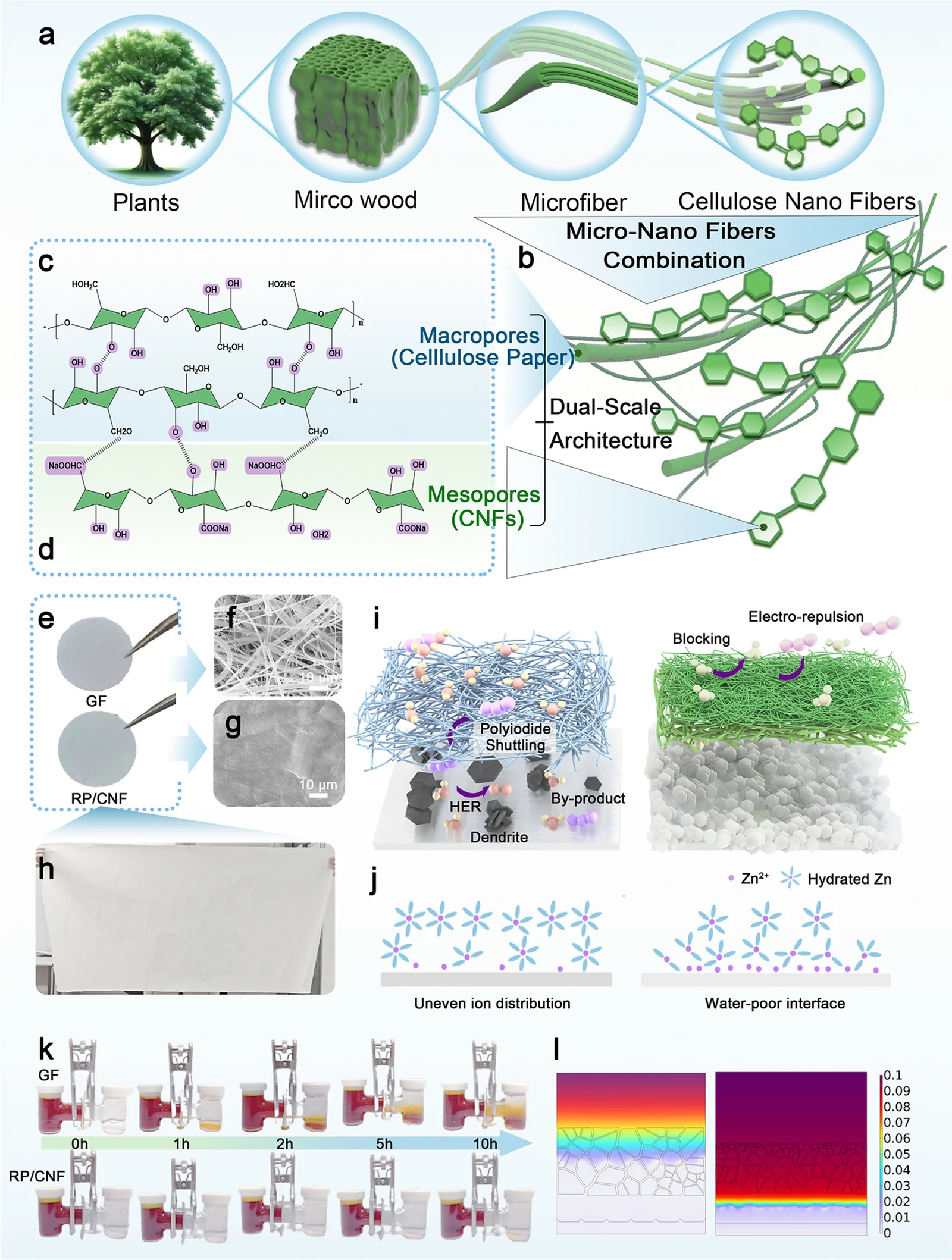
Competitive advantages of dual-scale asymmetric membranes. **a** Preparation of dual-scale cellulose membrane deriving from raw natural materials. **b** Illustration of cellulose nanofibers entangled with macrofibers. **c** Schematic illustration of macropore cellulose (rice paper) and **d** nanopore cellulose (CNFs). **e** Digital photographs of GF and all-cellulose membrane. SEM images of **f** GF and **g** cellulose membrane. **h** Asymmetric all-cellulose membrane with a size of 1.4 × 0.7 m^2^. Schematic illustration of GF and cellulose separator regulating the ion behavior **i** in bulk and** j** at interface. **k** Visualization experiments showing the inhibition of the polyiodide shuttling. **l** COMSOL simulation of ion concentration at the interface in GF and asymmetric membranes, respectively

As depicted in Fig. 1a, the synthesis of rice paper is derived from the plants, followed by process into microwood and microfiber bundles. For the production CNFs, further alkaline treatment is required to generate the functional groups terminated nanoscale cellulose. The dual-scale all-cellulose membrane is prepared via the direct pasting of CNFs slurry onto the rice paper (Fig. 1b). The Zn^2+^ diffusion mechanism within the dual-scale membrane is displayed in Fig. 1c, d. The CNF chains are interconnected via hydrogen bonds and further stabilized by Zn^2+^–COOH coordination, which facilitates directional Zn^2^⁺ transport. The Zn ions transport under the vast distribution of preferential absorption –COOH along dense nanopores rather than free migration within macropores in GF. Consequently, the abundant polar –COOH groups function as the ion valve for restricting the rampant 3D diffusion, inducing the ion-sieving effect for homogeneous plating.

The overall properties of the as-synthesized membranes are summarized in Fig. S4 to demonstrate the advantages of CNF-modified membranes. Inspiringly, the modified cellulose membrane offers a cost advantage over commercial glass fiber membrane while also being biodegradable and possessing higher mechanical strength (Fig. S5). In contrast to the commercial rice paper which features a macroporous structure, the as-prepared membrane is mesoporous and possesses ion-guiding capability (Fig. 1e–g). This structurally advantageous membrane can be inexpensively produced on a large scale, as shown by the digital photographs in Fig. 1h, which highlights its significant potential for battery applications. The detailed working mechanism is further schematically illustrated in Fig. 1i, j. Compared with conventional glass fibers, the dual-scale asymmetric membrane exhibits a collaborative functionality that creates a water-poor interface. This interface effectively suppresses side reactions and homogenizes ion distribution, thereby simultaneously mitigating hydrogen evolution reactions (HER), dendrite growth. Furthermore, the asymmetric membrane mitigates the severe capacity loss caused by the polyiodide shuttle effect through the ion-sieving action of its polar groups and the physical blocking of fibers. This is evidenced by the membrane’s stability, which shows no obvious color change after being left to rest for 10 h (Fig. 1k), which visually demonstrate the intrinsic blocking capability of the membrane against spontaneous diffusion. In addition to its ion-sieving capability, the functionalized membrane also regulates the interfacial concentration field, thereby optimizing the local electrochemical environment to mitigate side reactions (Fig. 1l). A comparative radar chart (Fig. S6) further summarizes the key performance metrics, underscoring the distinct performance advantages of the asymmetric all-cellulose membranes over their commercial glass fiber counterparts.

### Nucleation Models and Growth Behavior

**Fig. 2:**
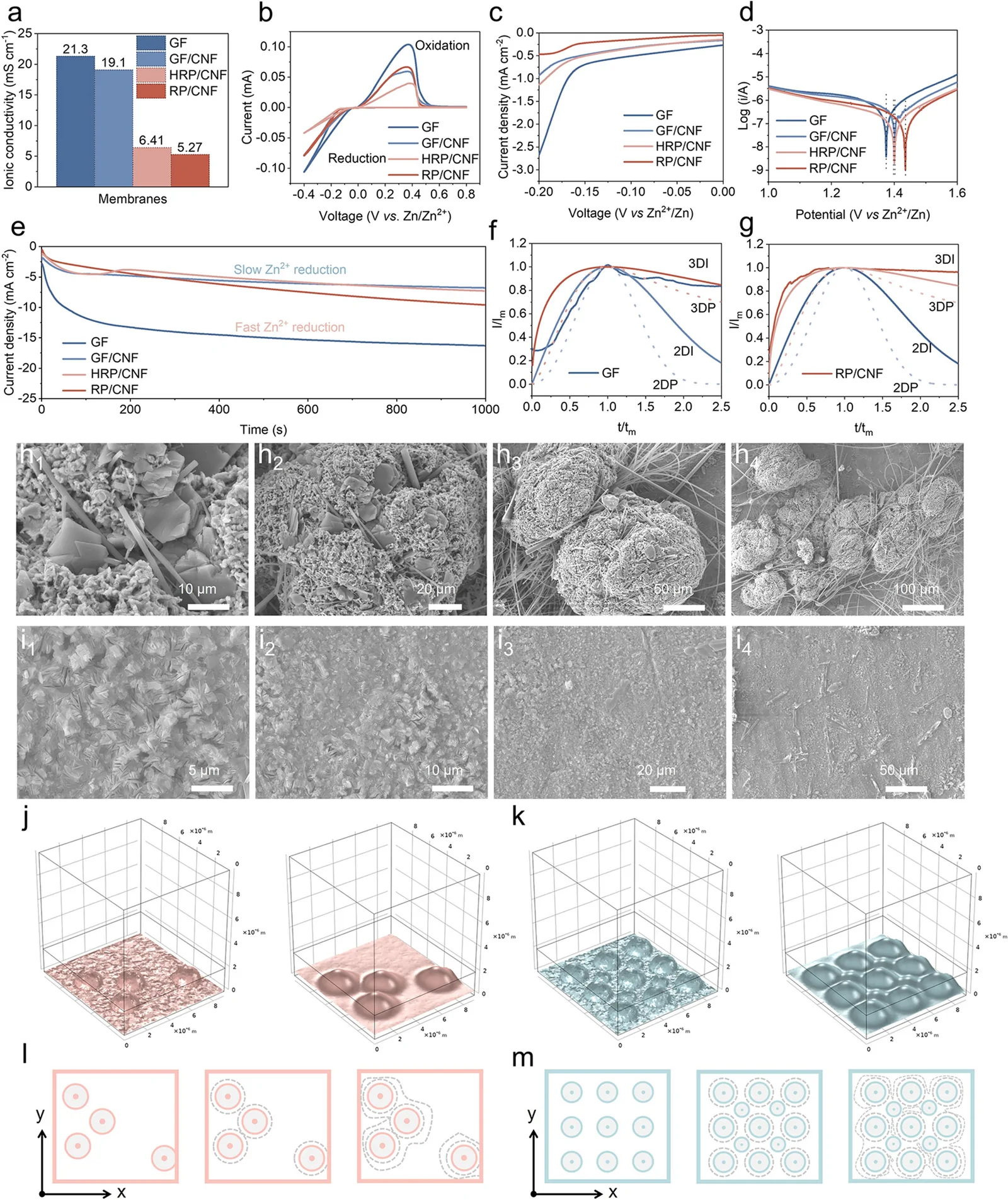
Investigating the nucleation models. **a** Ionic conductivity of respective membranes. **b** Cyclic voltammetry (CV). **c** Linear sweep voltammetry (LSV). **d** Plots of Tafel curves. **e** Chronoamperometry curves overpotential of − 150 mV for 500 s. SEM images of Zn electrode after plating with various magnifications in **h** GF and **i** RP/CNF. Theoretical 3D nucleation models of** f** GF and** g** RP/CNF. Initial nuclei models and final deposition models after 240 s of plating in** j** GF and **k** RP/CNF. Plane figures of the Zn nucleation and growth of **l** GF and **m** RP/CNF

The electrochemical properties of these membranes were compared to manifest the adaptability with Zn^2+^. As shown in Fig. 2a, the ionic conductivity of CNF-modified membranes all shows a decreased tendency, in which the GF/CNF slightly reduces while the rice papers sample dramatically decline (Fig. S7). This suggests that the CNFs would restrict the diffusion of Zn^2+^ due to reduced porosity compared to microporous structures of GF and raw paper. The EIS results of Zn||Zn cells with different membrane further reveal that the Zn^2+^ transport at the interphase is heavily affected by the functional CNFs due to the strong adsorption of polar groups (Fig. S8). The cyclic voltammetry (CV) confirms that the electrochemical activity of Zn^2+^ during plating or stripping was weakened as reflected by the shortened CV areas and the augmented nucleation potentials, which indicates that the localized ion concentration becomes homogeneous brought by this ion valve function of CNFs (Fig. 2b). The reduced Zn^2+^ reactivity is beneficial to limiting the tip effect and preventing the dendrite formation on Zn plate.

The functional membranes demonstrate obvious anti-HER ability, as displayed in LSV curves (Fig. 2c**)**. The onset potential for hydrogen evolution reaction significantly increases with the addition of CNFs. Apparently, water activity is locked by the polar –COOH groups, reducing the water-water bonding ability. The anti-corrosive capability is also important for the cycling stability of Zn||Zn cells as the formed by-products (ZHS) would harm the conductivity of Zn electrode. The higher Tafel potential and lower corrosion current mean greater stability and smaller corrosion rate where the bare GFs manifest the worse performances (Fig. 2d). The above results suggest that the CNF-modified separator can stabilize the interface of electrode/electrolyte to withstand adverse reactions.

The rampant growth of the Zn dendrite is closely related to the fast Zn^2+^ diffusion rate to the surface of Zn plate where the EDL exists. In addition, the Zn^2+^ reduction reaction occurred nearly instantly, and this creates an ion concentration gradient among bulk electrolyte, EDL and the Zn surface. The Zn^2+^ plating behavior is completely remodeled in CNF-modified membranes, as demonstrated by the chronoamperometry curve in Fig. 2e. The small current response reflects the low deposition rate, which further indicates the three-dimensional (3D) nucleation process of Zn^2+^ than that of GF membrane with 2D nucleation. The nucleation models can be further differentiated using current–time transient to compare with 2D Bewick, Fleischman and Thirsk (BFT) models and 3D Scharifker–Hills model [38–40]. The nucleation of Zn^2+^ occurs in two different paths that are the instantaneous (*I*) or progressive (*P*), determined by whether new nuclei form instantaneously during early stage or develop progressively over time [38, 39]. As indicated by Fig. 2f, it is obvious that the nucleation behavior in bare GF follows the mixed models between 3DP or 2DP during *t* < *t*_*m*_ showing that nucleation sites formed randomly and ceased to nucleate once the growth of nuclei. During *t* > *t*_*m*_, the Zn^2+^ deposits in a 3DI behavior under limited nuclei, thereby resulting in “tip effect.” Alternatively, the nucleation behavior exhibited by the GF/CNF conforms to a relatively different model that can be described as 2DI or 3DI during nucleation and 3DI growing model (Fig. S9). Furthermore, the nucleation demonstrated by the RP/CNF in Fig. 2g aligns with 3DI model, signifying that nucleation sites were instantaneously activated with smaller nuclei size and grew with even ion concentrations. It can be inferred that polar CNFs can promote instantaneous 3D diffusion-controlled Zn^2+^ nucleation and growth.

To gain deeper insights into the post-nucleation growth process, finite element simulations were performed using COMSOL Multiphysics. Hemispherical shapes were utilized to replicate the structure of Zn nuclei, as observed in SEM images of various electrodes after plating (Figs. 2h, i and S10). The CNF-modified separators display all uniform deposition morphology rather than dendritic surface with large sphere-like particles distributed in GF electrode. The initial model, shown in Fig. 2j, k, demonstrates that Zn nuclei of different sizes are uniformly distributed across the Zn plate, whereas block-like deposits appear randomly on the surface of bare Zn. Afterward, it shows a clear tendency to direct Zn^2+^ toward the Zn nuclei, a phenomenon that is more evident in bare Zn due to the scarce nucleation sites. As the simulation progresses, Zn^2+^ ions accumulated on the existing Zn nuclei along the path defined by the current vectors, enhancing the likelihood of localized deposition and leading to significant protrusions. Conversely, the plentiful Zn nuclei on the CNFs enhanced Zn plate interact with the electric field and ion flow, reducing the unevenness of Zn growth (Fig. 2l, m). Further deposition gradually filled in the spaces between nuclei, resulting in a smoother surface, which aligns with morphological observations.

### Electrochemical Performances and Identification of SEI Content

**Fig. 3:**
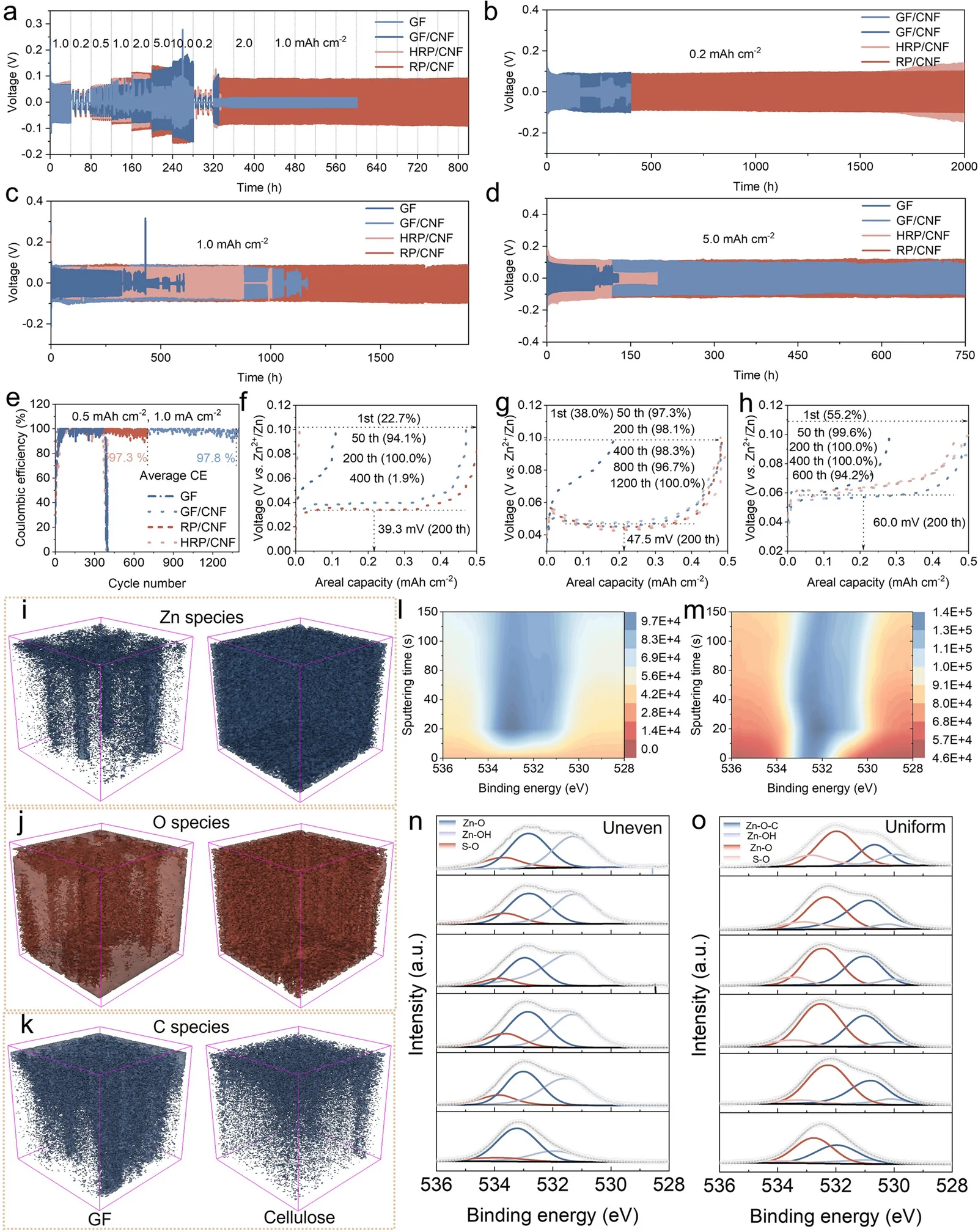
Electrochemical performance in symmetric cells. **a** Rate capability of GF, GF/CNF, HRP/CNF, and RP/CNF membranes, respectively. Potential-time curves under different current densities of **b** 0.2 mA cm^−2^, **c** 1.0 mA cm^−2^, and **d** 5.0 mA cm^−2^. **e** Plating/stripping CE. Voltage profiles of Zn//SS asymmetric cells at 0.5 mA cm^−2^/2.0 mAh cm^−2^ of **f** GF, **g** GF/CNF, and **h** RP/CNF. 3D TOF–SIMS results of zinc anode after plating with **i** Zn species, **j** O species, and **k** C species. Depth profile of zinc anodes with couture plots of O 1*s* and the corresponding XPS spectra in **l, n** GF and **m** GF/CNF

The cycling stability of Zn anodes in Zn||Zn cells was evaluated with different membranes. Figure 3a depicts plating/stripping curves of GF, GF/CNF, RP/CNF, and HRP/CNF at various current densities with a real capacity of 1.0 mAh cm^−2^. The cells were activated at 1.0 mA cm^−2^ before entering the rate cycle and apparently the pure GF cells fail to withstand even at 0.2 mA cm^−2^. Even though it remained stable at 1.0 mA cm^−2^, the irregular and limited nuclei formed during this stage continued to deposit afterward, causing uncontrollable growth of dendrites and battery failure. With the incorporation of CNF-based membranes, the rate capability was significantly improved, in which RP/CNF outperformed and even sustained nearly 500 h at 2.0 mA cm^−2^ after rate cycle. The long-term cycling performance is assessed at the current densities of 0.2 and 0.5 cm^−2^ with the areal capacities of 0.2 and 0.5 mAh cm^−2^, both demonstrating a remarkable stripping/plating stability of 2,000 h in the cells of RP/CNF in great contrast to bare GF of 156/136 h, respectively (Figs. 3b and S11). The RP/CNF further attained over 1,900 h reversibility at 1.0 mA cm^−2^ while bare GF only achieve ~ 320 h reversibility, confirming the effectiveness of suppressing dendrite formation (Fig. 3c). The high-rate capability over extensive cycling was also tested at the current density of 5.0 mA cm^−2^. As shown in Fig. 3d, the respective membranes achieve 87, 750, 116, and 750 h in GF, GF/CNF, HRP/CNF, and RP/CNF before short-circuit of batteries. Similar performance elevation was also observed at 2.0 mA cm^−2^ (Fig. S12), which proves that CNFs are beneficial to the homogeneous ion concentration for stable deposition.

To investigate the plating/stripping efficiency of Zn anodes, Zn||SS half cells were assembled in 2 M ZnSO_4_ at 1.0 mA cm^−2^ with areal capacity of 0.5 mAh cm^−2^. The initial CE values are 22.8%, 38.0%, 55.5%, and 24.4% for GF, GF/CNF, HRP/CNF, and RP/CNF membranes and the CE climbed slowly approaching 100%. However, the GF membrane exhibits significant fluctuations after just 300 cycles (Fig. 3e), with Coulombic efficiency (CE) values dropping below 20.0% along with HRP/CNF. In contrast, the GF/CNF and RP/CNF groups show a much more stable and reversible Zn plating/stripping process over 1,250 and 700 cycles, characterized by a higher average CE over 97.8% and 97.3%, respectively. These findings indicate that the CNFs separators outperform the GF separator in improving the reversibility of Zn plating/stripping.

Furthermore, the polarization potential shows an increasing trend from 39.3 mV (GF), 47.5 mV (GF/CNF), 60.0 mV (RP/CNF) to 38.9 mV (HRP/CNF), as displayed in Figs. 3f, g and S13. According to the classic nucleation theory, the size of nucleus is associated with the overpotential or [41], which can explain the instantaneous formation of nuclei with uniform and small particle size in CNFs based membranes. The additional nuclei in GF emerged during progressive nucleation either on the substrate or atop deposited dendrite. The formation of new nuclei competed with subsequent growth of Zn^2+^, resulting in rampant deposition and uneven morphology. The detailed deposition process is illustrated in Fig. 3i, j. Interestingly, when this functional nanocellulose was integrated with commercial wood pulp tissue to fabricate a composite all-cellulose membrane, it could effectively facilitate ion transport and inhibit zinc dendrite formation, showcasing the nanocellulose’s versatility across different hybridizations. Remarkably, the symmetric cell demonstrates long cycling time at various current densities of 0.2, 1.0, and 2.0 mA cm^−2^ that exceeds 230, 500, and 260 h, respectively (Fig. S14).

The difference of interfacial content on zinc plate after plating/stripping could result in the opposite effect on the stability of anode. The TOF–SIMS was performed to analyze the species of SEI under 3D distribution. From the 2D element distribution mapping, the electrode from GF membrane exhibits irregular growth of S and O species, indicating the random formation of by-product Zn_4_(OH)_6_SO_4_·xH_2_O (ZHS). With guidance of functional CNFs, homogeneous distribution is achieved for every component of the SEI (Fig. S15). A more direct proof can be found via 3D imaging as depicted in Figs. 3i, j and S16. The distribution of ZHS is ununiform and the Zn ions preferentially deposit along the orientation of the ZHS. This suggests that side reactions predominate, severely impeding the efficient deposition of zinc ions. Moreover, the C species is found to be more concentrated in the GF membrane, which is the result of CO_2_ reacting with the intermediate such as Zn(OH)_2_ due to corrosion reactions (Fig. 3k). On the contrary, the C species is homogeneously distributed in the RP/CNFs membrane, suggesting that the SEI forms accompanied by Zn deposition. Therefore, the above analysis shows that the cellulose-based membrane not only realize dendrite-free surface but also induce gradient SEI content, generating a robust and reversible interface.

Detailed interfacial SEI content was further determined using XPS depth profile. The dimensional plots of O 1*s* spectra from two different samples are presented in Fig. 3l, m. The peak intensity and the binding energy remain stable with the increasing sputtering time in the GF membrane, which indicates that the interfacial content mostly consists of specific oxygen-rich complex. Differently, the binding energy from cellulose modified membrane demonstrates an obvious shift to smaller values with broaden peaks, implying that the interfacial content evolves in a gradient tendency following the increase of etching depth. The deconvoluted spectra of O 1*s* can be identified with three peaks, S–O, Zn–O, and Zn–OH, which is ascribed to the following species including ZHS and ZnO. It can be observed that the interfacial content of zinc anode from GF membrane is mainly attributed to ZnO and ZHS. This manifests that the corrosion and side reaction occurred synchronously to form insulating layers, hindering the charge transfer and Zn^2+^ diffusion. In contrast, in RP/CNFs membrane, the Zn–OH diminishes another signal Zn–O–C arises through sputtering, signifying the effective suppression of side reaction. The shift of binding energy to higher value in C 1*s* spectra (Fig. S17) also proves that the gradient co-existence of Zn–O–C species derived from O-containing functional polar groups upon different plating depths, consistent with the TOF–SIMS results. Furthermore, this shift indicates that the CNF-modified interface possesses higher conductivity and better electrical contact with the Zn substrate compared to the uneven, highly resistive SEI formed on the GF sample.

### Identifying the Ion-sieving Effect of Asymmetric Membranes

**Fig. 4:**
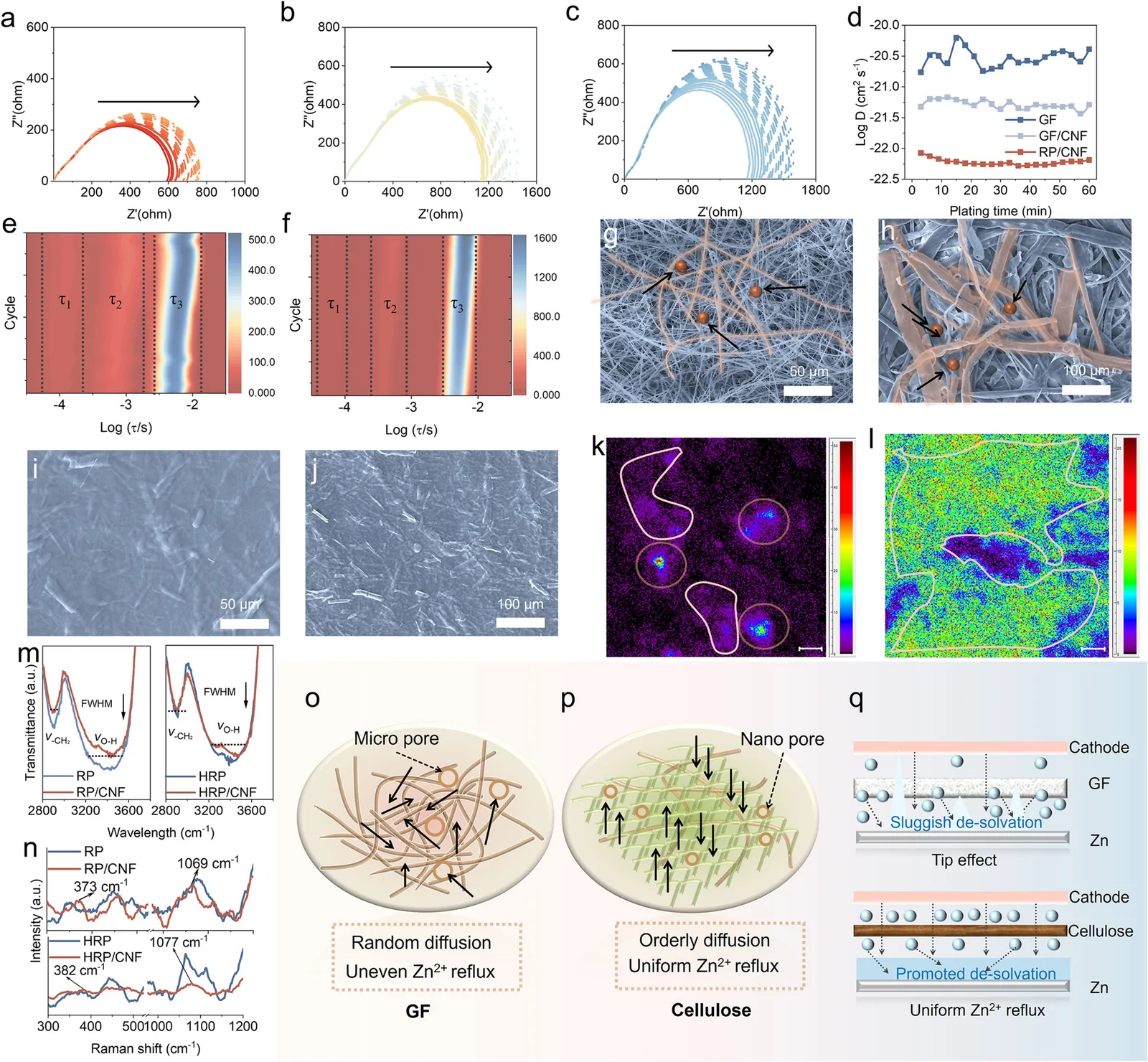
Dissecting the dual-scale architecture with interfacial stabilization. Nyquist plots of various cycles during plating in Zn||Zn of **a** GF, **b** GF/CNF, and **c** RP/CNF. **d** Diffusion coefficients of membranes during plating. DRT curves of **e** GF and **f** GF/CNF membranes. SEM images with macroporous structures of **g** glass fiber and **h** rice paper. SEM images with dense structures of **i** glass fiber and **j** rice paper. 2D TOF–SIMS images showing Zn element distribution in **k** glass fiber and **l** rice paper, respectively. **m** FTIR spectra and **n** Raman spectra. Illustration of Zn^2+^ concentration at the interface in **o** glass fiber and **p** dual-scale cellulose paper. **q** Illustration Zn^2+^ deposition at the interface

Electrochemical impedance spectroscopy (EIS) was performed to analyze the ion diffusion in the as-prepared membranes. All cells with GF, GF/CNF, and RP/CNF demonstrate increasing impedances as plating time increases. This suggests that the Zn nanoparticles subsequently deposited over the formed nucleus, amplifying the interface resistance (Fig. 4a–c). The EIS curves were further simulated using the equivalent circuit model in Fig. S18a. The CNF-modified membranes show much lower value of Zn^2+^ diffusion coefficients than bare GF, which is attributed to the ion reflux limiting caused by nanopores from interconnected fiber frameworks (Fig. 4d).

To gain deeper insight into the Zn^2+^ diffusion behavior, the distribution of relaxation times (DRT) analysis was employed to deconvolute the electrochemical impedance spectroscopy (EIS) results. This technique enables the isolation and identification of individual polarization processes without prior assumption of equivalent circuit models, offering a clearer perspective on interfacial dynamics. The normalized DRT profiles are shown in Fig. S18.

For the glass fiber (GF) separator, the position of the τ_1_ peak remained stable during the initial 10 cycles, indicating rapid solid-electrolyte interphase (SEI) formation (Fig. 4e). However, upon continued plating, the corresponding relaxation time exhibited a marked upward shift, suggesting progressively sluggish ion transport. This behavior is largely attributed to the accumulation of inactive “dead Zn,” which increases mass transfer resistance and locally aggravates the tip‑induced deposition effect. In contrast, the τ_1_ peak position for the GF/CNF membrane shows only minimal variation (Fig. S18b), implying that the slight increase in relaxation time was more likely related to stable SEI maturation rather than irreversible zinc deposition. This is consistent with reported observations that such variations are considerably smaller than those induced by “dead Zn” formation [42]. A similar stable trend was also observed in the RP/CNF membrane (Fig. 4f). The corresponding integrated impedances for each process (Fig. S18c–e) further revealed that although the cellulose‑based membranes exhibited higher overall impedance, the reaction kinetics were significantly improved. While the macroporous GF separator allows for unimpeded yet random ion diffusion, the CNF-based membrane enables a more efficient, surface-guided Zn^2+^ transport via a continuous Zn^2+^–COO^−^ hopping pathway. This results in a higher effective ion-transport rate (larger diffusion efficient D, shorter *τ*_3_) despite the smaller physical pore size. Coupled with a stabilized charge-transfer interface, this unique transport mechanism ensures a uniform ion flux that fundamentally suppresses dendritic growth.

The asymmetric all-cellulose membrane capitalizes on its structurally denser, nanoporous top layer to provide precise ion-sieving capabilities, a feature absents in the isotropic macroporous networks of glass fiber separators. This dual-scale architecture is pivotal in regulating Zn^2+^ flux, rather than allowing concentrated ionic reflux through free, unimpeded pathways. The membrane enforces a hierarchical transport mechanism, which can be seen in the bare glass fiber and rice paper (Fig. 4g, h). Such progressively tuned pore structure ensures uniformly distributed Zn^2+^ migration pathways. By preventing localized high ion concentrations, the membrane effectively suppresses the random nucleation of Zn^2+^, the notorious growth of dendrites, and harmful side reactions like HER that stem from sluggish de-solvation. This mechanism is directly visualized in Fig. 4i, j, where the cellulose membrane demonstrates a clear suppression of localized accumulation of Zn^2+^, attributable to its structural uniformity. The improved homogeneity of the membrane is further corroborated by 3D laser scanning microscopy (Figs. S20–S22), which reveals a dense and homogenous surface morphology. This dense yet uniform structure is critical for guiding uniform Zn^2+^ deposition and mechanically withstanding dendrite penetration.

The membrane exhibits an asymmetric structure that is both morphological and chemical in nature, collectively regulating interfacial behavior. The dense nanocellulose layer exhibits weak but significant interactions with free H_2_O and SO_4_^2−^ via hydrogen bonding and electrostatic repulsion. This dual-scale functionality of pore size directly regulates zinc deposition behavior. 2D TOF–SIMS mapping of the zinc electrode after cycling reveals scarce and uneven nucleation sites on bare zinc, which serve as the origin of dendritic growth (Fig. 4k). In sharp contrast, the CNF-modified electrode exhibits a large, continuous area of effective deposition, as highlighted in yellow (Fig. 4l), underscoring the critical role of the densely distributed, negatively charged groups in guiding uniform nucleation. Spectroscopic data confirm this improved uniformity. The narrowed *v*_O-H_ peak in FTIR (Fig. 4m) indicates a more homogeneous environment, resulting from the robust H-bond network. Similarly, the sharpened C–O–C peak in Raman spectra (~ 1077 cm^−1^, Figs. 4n and S24) reflects a highly ordered structure. This remarkable interfacial stability stems directly from the membrane’s synergistic dual-scale design (Fig. 4o, p). The abundant carboxyl (–COOH) groups, highly concentrated within the functional layer, coordinate with Zn^2+^ ions to create a continuous, directional ion-hopping pathway. This ensures uniform ion transport and eliminates the tip effect that accelerates dendrite growth. (Fig. 4q). Concurrently, the graded hydrogen-bonding network effectively immobilizes free water molecules, thereby mitigating side reactions and ensuring exceptional interfacial stability during prolonged cycling.

### Simulating the Zn^2+^ Sieving Behavior of CNFs

**Fig. 5:**
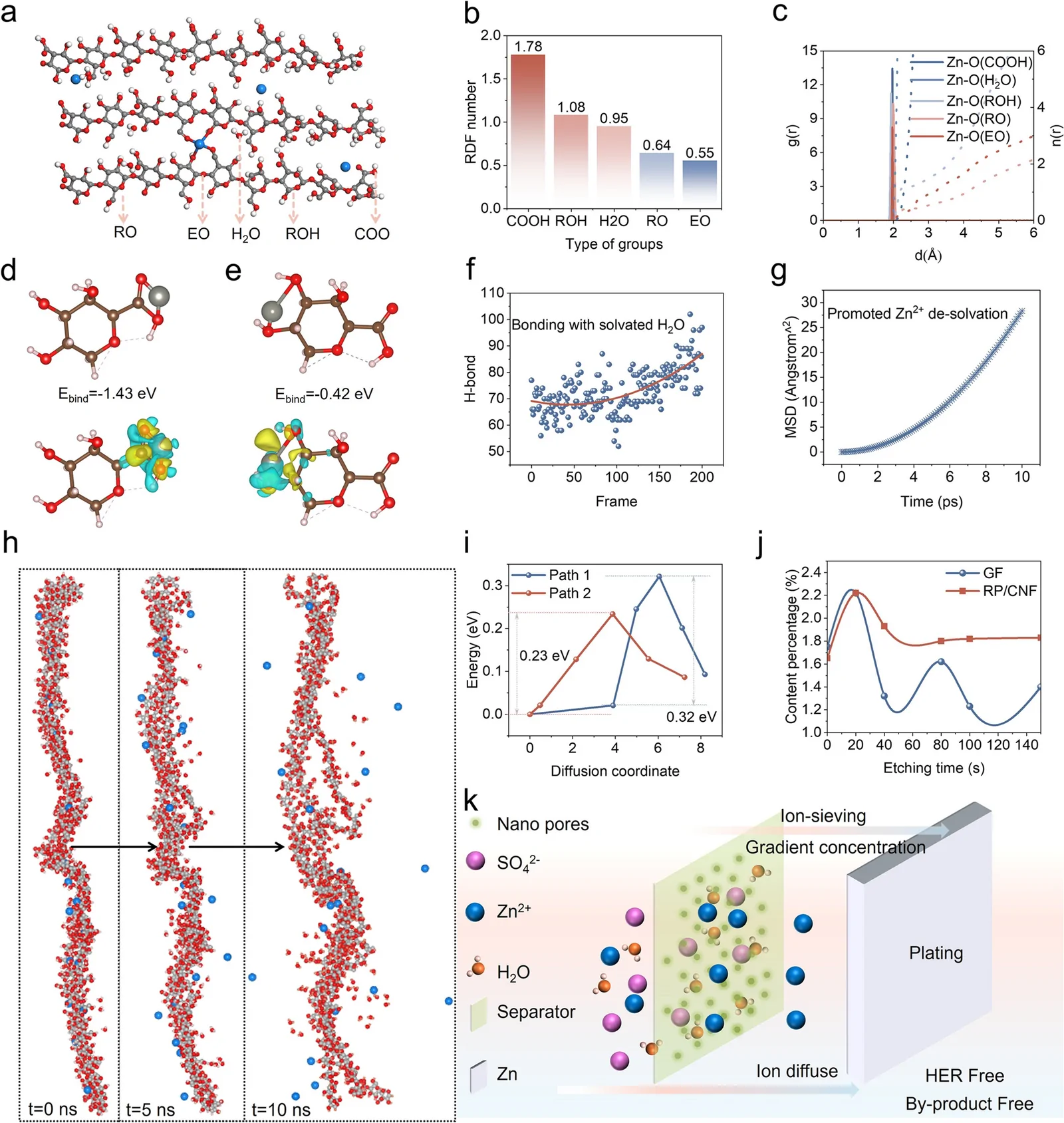
Zn^2+^ transport mechanism in CNFs chains. **a** Diagram showing the chemical environment of CNFs framework with different types of oxygen atom. C (gray), H (white), O (red), and Zn (blue) atoms, respectively. **b, c** RDF number plotted from MD simulation. The adsorption energy of Zn^2+^ to polar groups of **d** -COOH and **e** -OH. **f** Dynamic change in number of H-bond during Zn^2+^ transport. **g** Zn^2+^ diffusion coefficient. **h** Structural snapshots from MD simulations of numbers of Zn^2+^ and H_2_O transporting through CNF chain within 5 ns. **i** Energy barrier under diffusion path and **j** Atomic percentage of S content. **k** Mechanism of ion sieving of cellulose membrane

The incorporation of CNFs into paper as separating membrane manifests exceptional elevation in electrochemical performances, which is significantly impacted by the molecular structure of CNFs demonstrated in Fig. 5a. Distinctly, different oxygen-containing functional groups including hydroxyl (R–OH), carboxylate (COO^−^), alkoxide (RO^−^), and ether (E–O) moieties exist in the CNFs interconnected network, exerting individual coordination effects on positively charged Zn^2+^ given their different polarity, which was determined by molecular simulation (MD). According to the RDFs number in Fig. 5b, they all have similar bond length around 2.0 Å, signifying that the functional groups can have close interaction with Zn^2+^ at the interphase. Expectedly, the two most polar groups Zn^2+^–O (–COOH) and Zn^2+^–O (–OH) possess high RDF coordination numbers with 1.78 and 1.08, respectively, adding up to 2.86 significantly larger than that of Zn^2+^–O (H_2_O) with 0.95 (Fig. 5c). In addition, the RDF of less polar groups Zn^2+^–O (R–OH) and Zn^2+^–O (EO) also indicate improved coordination ability. The RDFs result proves that the solvated Zn^2+^ will be absorbed within the CNFs channels because of the strong interaction. The detailed absorption is unveiled by the binding energies of Zn^2+^ solvation configurations based on the density functions theory (DFT) calculations. From the results in Fig. 5d, e, [(R–COOH)Zn] shows a much lower binding energy of − 1.43 eV than [(R–OH)Zn] with − 0.42 eV, which explains that the –COOH is the preferential adsorption sites for Zn^2+^, leading to fast transport and de-solvation. The uneven distribution of polar group reveals that the Zn^2+^ transport via hopping from one polar site to another.

The H-bond environment among functional groups and H_2_O was also detected during Zn^2+^ migration. The hydrophilic CNFs can generate internal force via H-bond among polymer chains, thus forming interconnected membrane network. The free water molecules can be immobilized to lower localized content for reducing water-related side reactions. As shown in Fig. 5f, initially H-bond number maintained dynamic equilibrium before 100 frames, indicating a stable diffusion process of water molecules following the open molecular channels. After that, it shows a slow increasing process, which can be attributed to progressively de-solvating of hydrated shells. Further evidence is provided in Fig. 5g where the diffusion coefficient is synchronously simulated. The result visually demonstrates a diffusion accelerating behavior of Zn^2+^ where the solvated Zn undergoes step by step de-solvation of H_2_O. This increases the transport speed with smaller solvated cluster within the cellulose channels, which promotes instantaneous nucleation as indicated by current–time transient curves.

Figure 5h illustrates the simulated dynamic motion of respective electrolyte components during plating with system structures shown in Fig. S25. Distinctly, the Zn^2+^ diffused along the molecular polymer channels while the backbone of cellulose chains barely moved, suggesting that the Zn^2+^ could migrate in the way of hopping that decouples from polymer chains moving. The Zn–O coordination and the bound water that generated open channels for Zn^2+^ within CNFs framework, could probably support the Zn^2+^ hopping mechanism within cellulose chains. Interestingly, the unbound water exhibited a tendency to migrate in the opposite direction to Zn ion. This proves that the free water can be fixed locally to further facilitate de-solvation and lower the water content of Zn^2+^ clusters. It was observed that Zn ions migrating along nanocellulose chains encounter different energy barriers. Path 1 presents an electronic energy barrier of 0.32 eV, whereas Path 2 has a lower barrier of 0.23 eV (Figs. 5i and S26). This unveils that Path 2 is more favorable for Zn^2+^ transport owing to its reduced resistance, potentially enhancing Zn^2+^ transfer kinetic at the electrolyte/electrode interphase. The content of ZHS by-product is associated with the concentration of SO_4_^2−^, which is reflected by atomic percentage from S 2*p* spectra (Figs. 5j and S27). The content in RP/CNF shows a uniform value as the depth changes. This is in great contrast to GF with unstable S 2*p* vales, indicating the uneven distribution of ZHS. As illustrated in Fig. 5k, the ion-sieving mechanism is driven by the nanoporous structure of cellulose nanofibers (CNFs) and the strong interaction between their carboxyl (–COOH) groups and Zn^2^⁺ ions, which selectively facilitate Zn^2^⁺ transport while restricting anion and water molecule migration. This selective ion transport reduces the 3D diffusion of Zn^2^⁺, enabling uniform ion distribution and minimizing dendrite formation. Furthermore, the confined pore channels and electrostatic interactions promote efficient Zn^2^⁺ nucleation and growth, enhancing overall plating and stripping efficiency.

### Use of Cellulose Paper Membranes in Full Cells

**Fig. 6:**
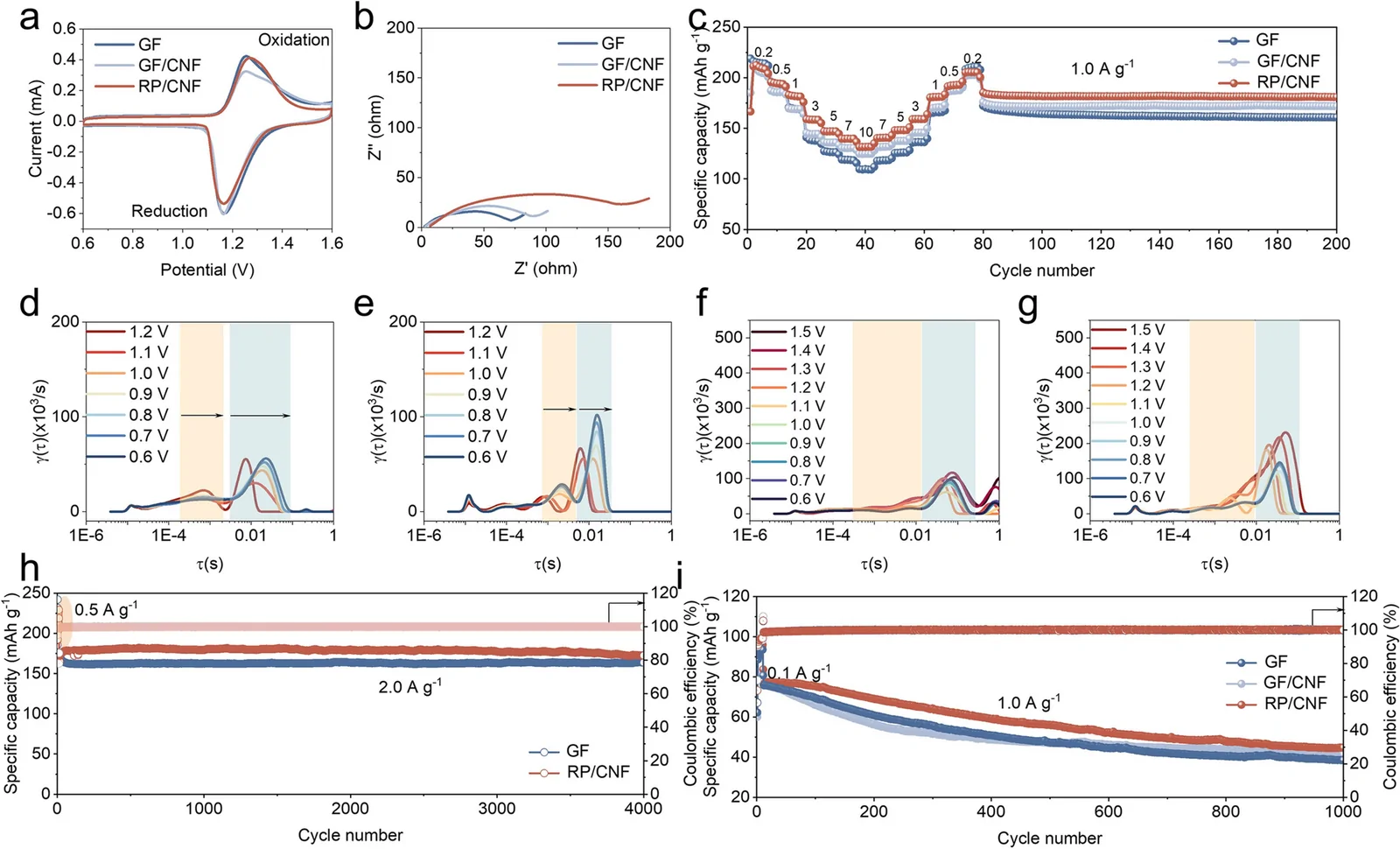
Evaluation of full cells performances when compacted with I_2_ and PANI cathode. **a** CV curves at the scanning rate of 0.2 mV s^-1^. **b** Nyquist plots of GF, GF/CNF, and RP/CNF membranes. **c** Rate capability at various current densities. DRT analysis at the initial discharge process with **d** GF and **e** RP/CNF and second discharge process with **f** GF and **g** RP/CNF. **h** Cycling life-span in Zn||I_2_ at 2.0 A g^-1^. **i** Cycling life-span in Zn||PANI at 1.0 A g^-1^

The electrochemical performances of full batteries were further evaluated in Zn||I_2_ configuration to evaluate the practicability. As shown in Fig. 6a, the cyclic voltammetry (CV) profiles show well-defined redox peaks with overlapping curves for the GF and modified separators. This suggests that the reversible reactions of Zn/Zn^2+^ was not affected by the micropores formed by the cellulose nanofibers. However, RP/CNF shows a slight polarization of anodic peak with increased oxidation potential, which is also reflected by the Nyquist plots from Fig. 6b of the modified separators exhibiting a larger charge-transfer resistance, indicating the restricted ion diffusion through the tailored pore structure. Consequently, the reduced localized current at the interface caused by the restricted ion diffusion within the interconnected pore structure could compromise the reduction reaction of I_2_ to I^−^ and thereby decrease the discharge capacity in the initial cycles. The charge–discharge curves in Fig. 6c were analyzed to evaluate the rate capability of respective membranes. In detail, the GF membrane was expected to demonstrate a higher specific capacity in the first cycle with 219.0 mAh g^−1^ at 0.2 A g^−1^ while the CNF-modified membranes delivered 185.3 and 166.7 mAh g^−1^ for GF/CNF and RP/CNF, respectively. Similar amount of discharge capacities are also found when the current density is returned to 0.2 A g^−1^ but it also sustains at small current densities. Apparently, the Zn||I_2_ in RP/CNF membrane demonstrates superior rate capability at 0.5, 1.0, 3.0, 5.0, 7.0, and 10.0 A g^−1^, which is probably ascribed to reducing shuttle effect of polyiodide. Not only the polar groups (–COOH) could create electrostatic repulsion but also the CNFs form the confined pore channels to prevent random diffusion of iodine ions, which jointly contributes to eliminating the so called “shuttle effect.” As reflected by charge and discharge curve in Fig. S28, GF membrane clearly shows large voltage gaps between the charge and discharge plateaus, indicating sluggish kinetics and high internal resistance. Vividly, the RP/CNF battery exhibits much smaller voltage hysteresis and well-defined voltage plateaus under similar rate conditions. Furthermore, the cells withstood discharge–charge procedure over 120 cycles at 1.0 A g^−1^ after rate test. The above results demonstrate that this all-cellulose membrane has great potential as a commercial inexpensive battery separator.

Notably, the initially higher specific capacity observed for the GF separator at low current densities is attributed to an inflated apparent capacity from the parasitic polyiodide shuttle effect, which allows for repeated yet unsustainable redox cycling of shuttle species. In contrast, the RP/CNF membrane suppresses this shuttle from the beginning, delivering a slightly lower but intrinsic capacity. As the rate increases, the GF cell suffers from severe polarization and rapid failure due to uncontrolled dendrite growth and persistent shuttling. The RP/CNF separator, however, maintains a uniform ion flux and interfacial stability, enabling its superior high-rate performance and long-term cycling reversibility. This performance inversion underscores that the true merit of a separator lies not in transient initial capacity but in sustaining electrochemically reversible reactions under practical conditions.

The DRT analysis was further employed to gain insights into the ion-transport mechanisms. According to the reaction mechanism, the discharge process on cathode is divided into two gradual reduction steps where the absorbed I_2_ is firstly reduced as soluble I_2_^−^ and then I^−^, in which the I_2_^−^ reduction is the rate-controlled step. There are two main peaks corresponding to the above two reaction processes. As shown in Fig. 6d, the DRT curve indicates a noticeable shift at around *τ* ~ 0.01 s toward longer relaxation times for the GF membrane, which could be indexed to the reduction of soluble I_2_^−^ from insoluble I_2_. While RP/CNF manifests a much shorter time in both reduction steps, reflecting a more efficient transport pathway Fig. 6e. In the second discharge curves, an extra peak appears at *τ* ~ 1.0 s in GF membrane, and this could illustrate an occurrence of side reactions such as HER (Fig. 6f, g). This enhancement in ion-transport efficiency is likely due to the polar –COOH terminated CNFs increase electrocatalytic active sites for reversible I_2_/I^−^ and the strong repelling effect to polyiodide such as I_3_^−^, which discloses the ion-sieving effect of CNFs strengthen membranes (Fig. 6h). The long-term cycling performance was conducted to demonstrate the longevity of such multifunctional paper-based membranes. After cycling activation at 0.5 A g^−1^, the full cell reaches an ultra-stable cycling life over 4,000 cycles at 2.0 A g^−1^ with higher discharge capacity of 172.8 mAh g^−1^ than that of GF membrane (Fig. S29).

The versatility of cathode material was further extended to polyaniline (PANI) and the full-cell performance is shown in Fig. 6i. Apparently, both CNF-modified membrane GF/CNF and RP/CNF display elevated capacity output over 1,000 cycles. The analysis of evaluation of such asymmetric membrane shows the practical application potential in aqueous batteries system. To better demonstrate the performance of this paper-based membrane, key metrics such as cycling life, capacity retention, Coulombic efficiency, and rate capability are summarized in Table S1 for such separator alongside several state-of-the-art separators including hydrogel-based separators, inorganic-modified separators (e.g., silica nanosheet, halloysite nanotubes), MOF-functionalized separators, and other cellulose-based separators.

## Conclusion

In summary, an innovative all-cellulose separator featuring a deliberate asymmetric architecture is developed, which strategically couples a macroporous rice paper substrate with a nanoporous, carboxyl-functionalized cellulose nanofiber (CNF) top layer. This structural and chemical asymmetry is pivotal to its success, enabling synergistic ion regulation and interface stabilization that are unattainable with homogeneous separators. The CNF-rich functional layer establishes a dense network of Zn^2+^–COOH coordination sites, creating directed ion-hopping pathways that homogenize zinc flux and suppress dendrites. Simultaneously, the dual-scale pore-size structure from macroporous bulk to mesoporous surface combined with electrostatic repulsion, imposes a molecular sieving effect that selectively facilitates Zn^2+^ transport while restricting water mobility and anion shuttling. As a result, this symmetric membrane delivers exceptional cycling stability in Zn||Zn symmetric cells, high Coulombic efficiency in Zn||Cu configurations, and sustained capacity in Zn||I_2_ full cells, all while maintaining mechanical robustness and full biodegradability. This work highlights the transformative potential of asymmetric design in constructing low-cost, eco-friendly, and high-performance separators for ZIBs as a concept that can be extended to other metal-ion battery systems.

## Supplementary Information

Below is the link to the electronic supplementary material.

Supplementary file1 (DOCX 13630 KB)
