# Supplementary material for: Ion-Sieving Dual-Scale Asymmetric Cellulose Membrane as a Sustainable Paper-Based Separator for Ultra-Stable Zinc Anodes
Source: Nanomicro Lett. 2026 Mar 27;18:307. doi: 10.1007/s40820-026-02165-0 (PMC13031489; doi:10.1007/s40820-026-02165-0)
Supplement: Supplementary file 1 — Supplementary file1 (DOCX 13630 KB) [file 40820_2026_2165_MOESM1_ESM.docx]

Supporting Information for

**Ion-Sieving Dual-Scale Asymmetric Cellulose Membrane as a Sustainable Paper-Based Separator for Ultra-Stable Zinc Anodes**

Xinlong Liu^1,2^, Junze Zhang^1^, Cuiqin Fang^1^, Yana Xiao^1^, Yuejue Yang^1^, Shuai Wang^1^, Qingjun Yang^1^, Yaopeng Wu^1^, Bingang Xu^1,2^*

^1^Research Institute for Intelligent Wearable Systems, The Hong Kong Polytechnic University, Kowloon, 999077, Hong Kong, P. R. China

^2^Research Centre for Resources Engineering towards Carbon Neutrality, The Hong Kong Polytechnic University, Kowloon, 999077, Hong Kong, P. R. China

*Corresponding author. E-mail: [tcxubg@polyu.edu.hk](mailto:tcxubg@polyu.edu.hk) (Bingang Xu)

**S1 Methods**

**S1.1 Electrochemical Evaluation**

CR-2032 coin cells were fabricated in ambient conditions, utilizing 100 μm-thick Zn foil anodes and glass fiber (GF/A) separators. Galvanostatic charge/discharge (GCD) profiles for symmetric Zn||Zn cells, Zn||SS half-cells, and full Zn||I_2_/PANI cells were recorded on a NEWARE battery testing system. Cyclic voltammetry (CV), linear sweep voltammetry (LSV), Tafel analysis, chronoamperometry (CA), and electrochemical impedance spectroscopy (EIS) were conducted using a CHI 660E electrochemical workstation. In-situ EIS measurements were performed during Zn plating/stripping at 2.0 mA cm^-2^, with 3 min charge/discharge intervals and 1 min rest periods between cycles.

**S1.2 Material Characterization**

Morphological and elemental analyses were performed via field-emission scanning electron microscopy (FE-SEM, Tescan MIRA) coupled with energy-dispersive X-ray spectroscopy (EDS) and high-resolution transmission electron microscopy (HR-TEM, JEOL JEM 2100F). Crystallographic structures were examined using X-ray diffraction (XRD, Rigaku SmartLab 9kW), while chemical bonding states were probed via X-ray photoelectron spectroscopy (XPS, Thermo Fisher Nexsa). Functional groups in materials and electrolytes were identified using Fourier-transform infrared spectroscopy (FTIR, Thermo Scientific) across 450-4000 cm^-1^. Interfacial evolution on Zn electrodes was studied by grazing-incidence XRD (GIXRD, Bruker D8) with incident angles of 0.5°-2.6°, and crystallographic orientation was mapped via 2D wide-angle XRD (2D-WAXD, Rigaku Ultima IV). Molecular interactions in electrolytes were analyzed using Raman spectroscopy (Renishaw InVia, 532 nm laser).

**S1.3 Distribution of Relaxation Times (DRT) Analysis**

The DRT analysis was performed using the open-source package DRTtools to deconvolute the electrochemical impedance spectroscopy (EIS) data, which were measured under open-circuit conditions across a frequency range of 100 kHz to 10 mHz with a sinusoidal perturbation amplitude of 10 mV. The raw EIS data were first validated via Kramers-Kronig (KK) transformation to ensure consistency and compliance with linearity and stationarity assumptions. The DRT profiles were derived by solving the Fredholm integral equation through Tikhonov regularization, where the regularization parameter (λ) was optimized using the L-curve method to balance fitting accuracy and smoothness.

Key parameters included a relaxation time (τ) range spanning 10^-6^ to 10^3^ s, covering both fast and slow kinetic processes. The resulting DRT spectra were deconvoluted into distinct peaks, each corresponding to specific electrochemical mechanisms. The x-axis denotes the time constant (*τ*), calculated as the product of resistance (*R*) and capacitance (*C*), which is independent of surface area and indicates the intrinsic properties of each process. The peak area signifies the impedance of the corresponding process. Thus, processes with varying *τ* values can be distinguished and identified in the DRT plots. Importantly, changes in each process during cycling can be tracked and correlated with cell performance, such as capacity loss and overpotentials. Typically, slow processes are linked to mass transport in the bulk electrolyte, like Warburg diffusion; intermediate processes pertain to charge transfer reactions; and fast processes involve ion transport across the SEI layer. Apparently, there is only one Zn^2+^ diffusion behavior observed that occurs in the process of *τ*_3_, which could be ascribed to ion transport to the across SEI to the Zn nuclei. The SEI content could be derived from the immediate reaction such as zinc hydroxide sulfate (Zn_4_SO_4_(OH)6·xH_2_O) when soaked in electrolyte and corrosion products including zinc oxide (ZnO) during plating.

**S1.4 Calculation of Diffusion Coefficient**

The Warburg coefficient $\sigma$ was obtained from the linear region of the plot of the real part of the impedance ($Z^{'}$) against the inverse square root of the angular frequency ($\omega^{-1/2}$) in the low-frequency range (typically below 1 Hz). The slope of this linear fit corresponds to $\sigma$:

$$Z^{'}=R_{e}+R_{ct}+\sigma\omega^{-1/2}$$

where $R_{e}$ is the electrolyte resistance and $R_{ct}$ is the charge transfer resistance.

The diffusion coefficient was then calculated using the following equation [S1]:

$$D_{Zn^{2+}}=\frac{R^{2}T^{2}}{2A^{2}n^{4}F^{4}C^{2}\sigma^{2}}$$

Where *R* is the gas constant (8.314 J mol^-1^ K^-1^), *T* is the absolute temperature (298 K), *A* is the surface area of the Zn electrode (1.0 cm^2^), *n* is the number of electrons transferred per ion (2 for Zn^2+^/Zn), *F* is Faraday's constant (96485 C mol^-1^),*C* is the bulk concentration of Zn^2+^ in the electrolyte (2 mol L^-1^ = 2 × 10^-3^ mol cm^-3^).

**S1.5 Molecular Dynamics Simulation**

In this study, we employed the Materials Studio software to construct cellulose monomer models, which were subsequently polymerized to a degree of polymerization (DP) of 50. Three polymer chains were assembled, incorporating Zn^2+^ and H_2_O molecules at a ratio of 1:4, serving as the initial model for molecular dynamics (MD) simulations. The optimization of these polymer chains was conducted with convergence thresholds set at 0.001 kcal/mol for maximum energy change, 0.5 kcal/mol/Å for maximum force, and 0.015 Å for maximum displacement. MD simulations were performed at 298 K under the NVT ensemble with a time step of 10 fs and a total simulation duration of 10 ns, considering the first 1 ns as the equilibration phase. To accurately assess Zn^2+^ diffusion properties without thermostat interference, subsequent simulations were conducted under the NVE ensemble. Post-simulation, the models were analyzed for dynamic properties.

**S1.6 Theoretical Calculation**

The binding energies of Zn²⁺ with carboxyl (-COOH) and hydroxyl (-OH) groups on nanocellulose were calculated using the CASTEP module. The Perdew-Burke-Ernzerhof (PBE) form of the Generalized Gradient Approximation (GGA) was employed to describe electron interactions, while the Projector Augmented Wave (PAW) method was used for electron-ion interactions. The Monkhorst-Pack k-point grid was set to 3×3×1, with a cutoff energy of 400 eV. A vacuum layer of 10 Å was added to eliminate periodic effects along the Z-axis. During model relaxation, the maximum force convergence was set to 5×10^-2^ eV/Å, and the energy convergence was set to 1.0×10^-5^ eV/atom.

**Supplementary Figures**


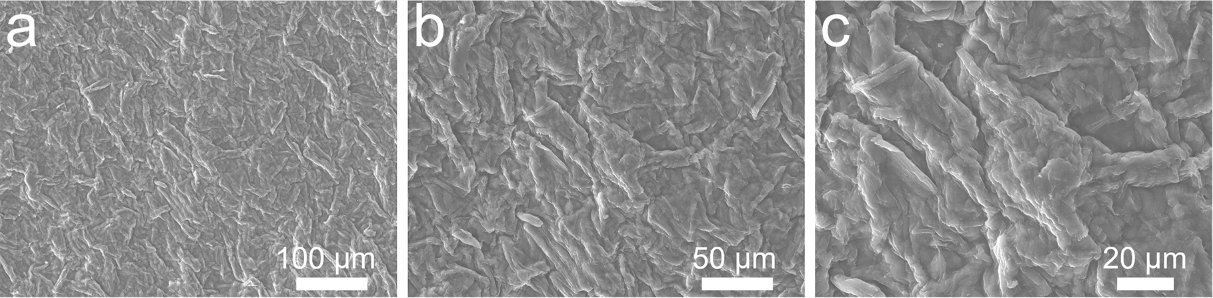


**Fig. S1** SEM images of as-prepared cellulose nanofibers (CNFs)


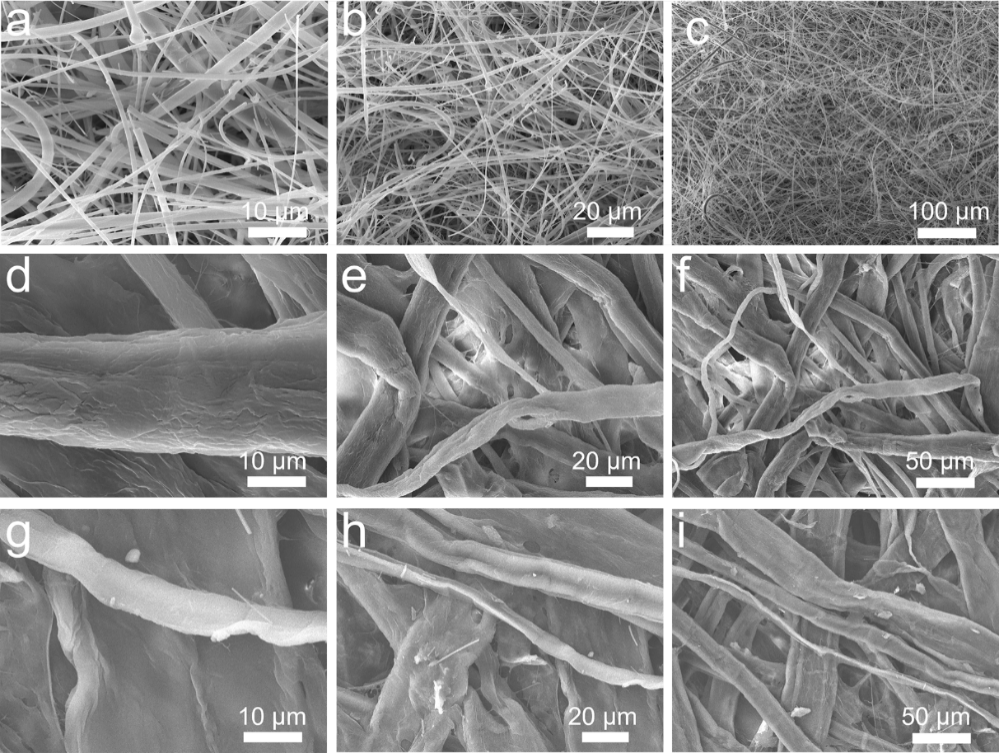


**Fig. S2** SEM images of **a-c**) glass fiber and naturally derived raw paper **d-e**) rice paper, **g-i**) half rice paper


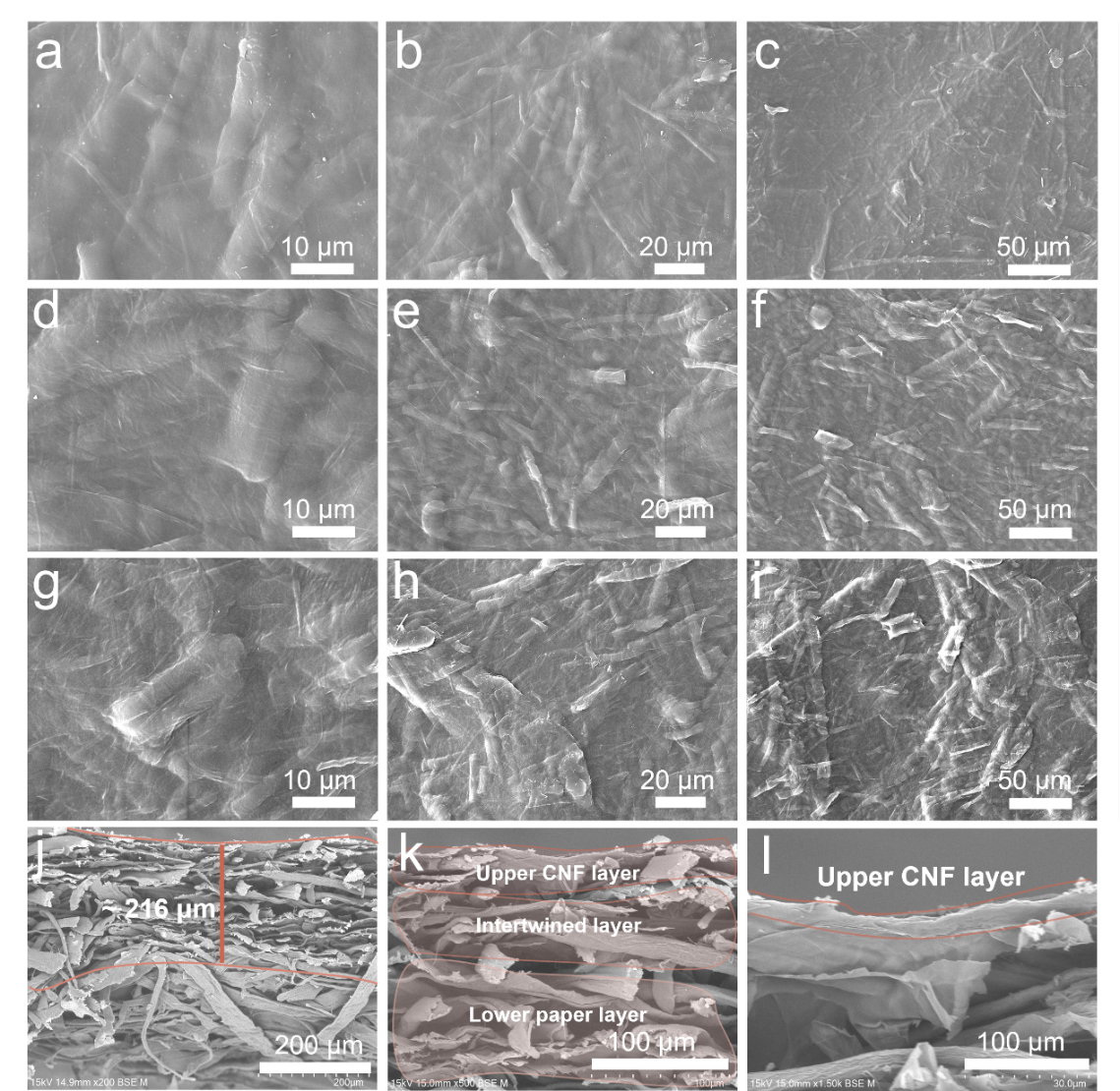


**Fig. S3** SEM images of as-prepared CNFs modified membranes: **a-c**) glass fiber, **d-f**) rice paper, **g-i**) half rice paper. **j-l**) Cross-section images of CNF integrated rice paper


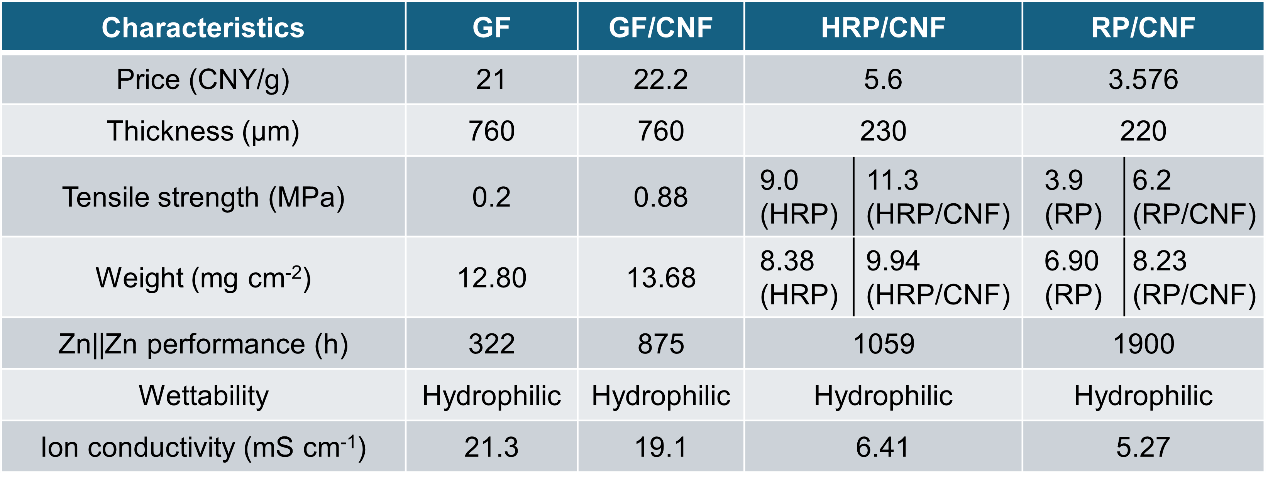


**Fig. S4** Comparison of physical and electrochemical characteristics between commercial glass fiber and cellulose membranes


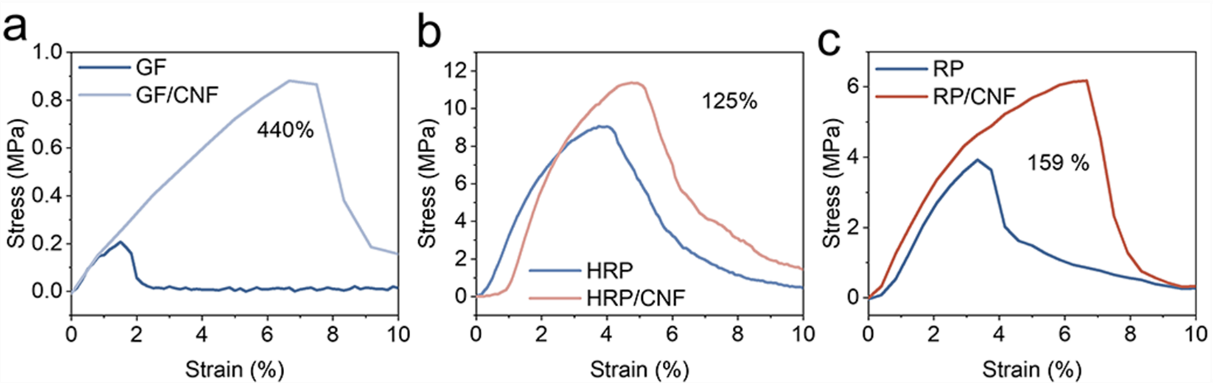


**Fig. S5** Tensile strength of as-prepared raw and CNF modified membranes with **a**) GF, **b**) RP, **c**) HRP


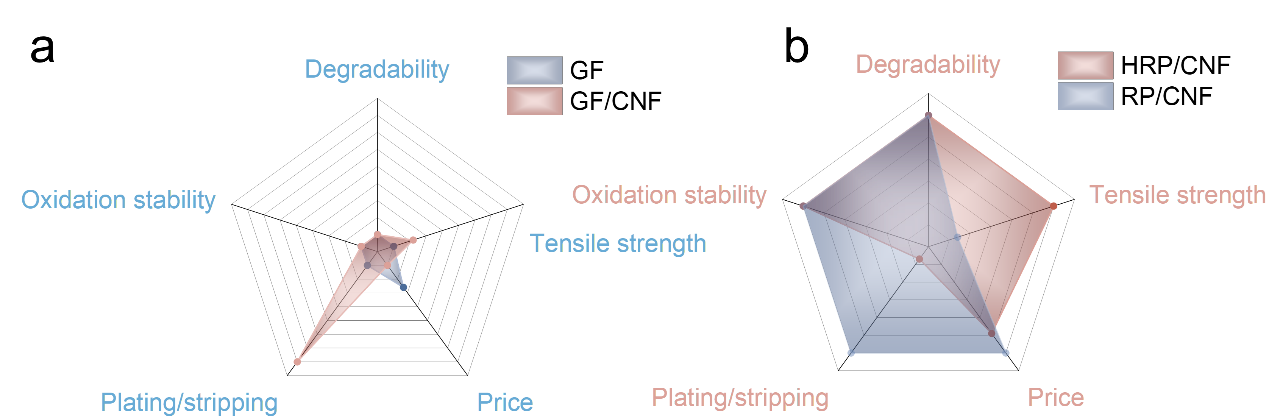


**Fig. S6** Radar charts of **a**) GF and **b**) CNFs membrane


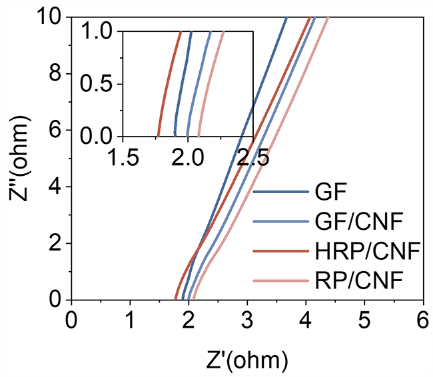


**Fig. S7** Ionic conductivities of respective membranes including GF, GF/CNF, HRP/CNF, and RP/CNF


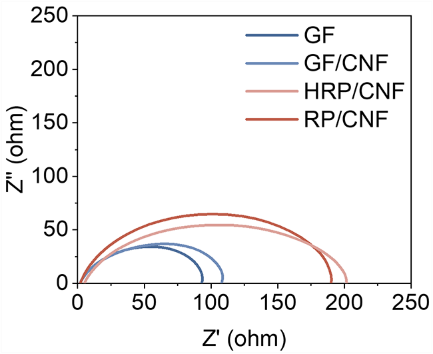


**Fig. S8** Nyquist plots of respective membranes including GF, GF/CNF, HRP/CNF, and RP/CNF in fresh Zn||Zn cells


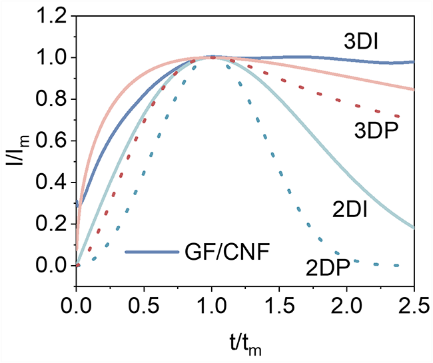


**Fig. S9** Theoretical 3D nucleation models of GF/CNF membrane


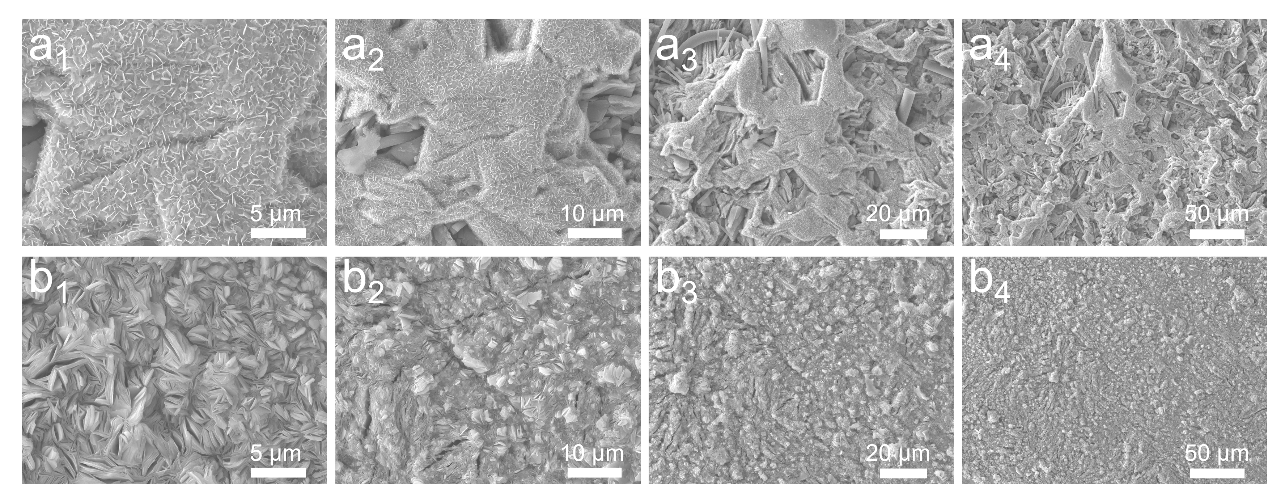


**Fig. S10** SEM images of Zn electrodes after cycled within different membranes: **a**) GF/CNF and **b**) HRP/CNF


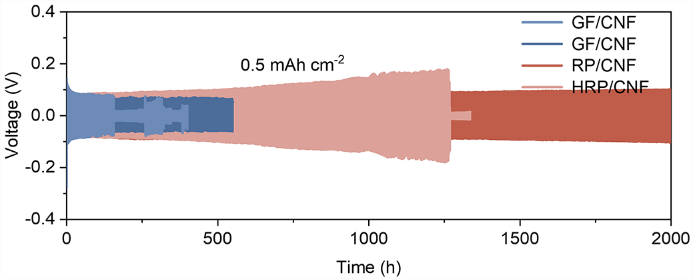


**Fig. S11** Potential-time curves under 0.5 mA cm^-2^ with membranes including GF, GF/CNF, HRP/CNF, and RP/CNF


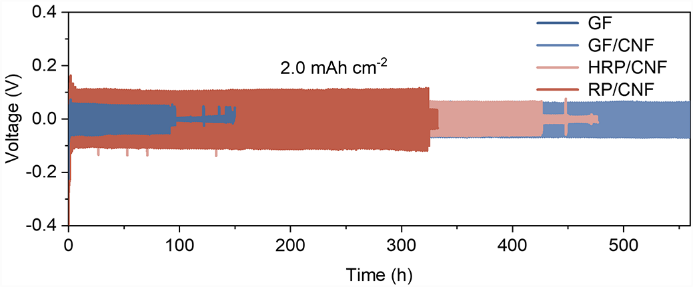


**Fig. S12** Potential-time curves under 2.0 mA cm^-2^ with membranes including GF, GF/CNF, HRP/CNF, and RP/CNF


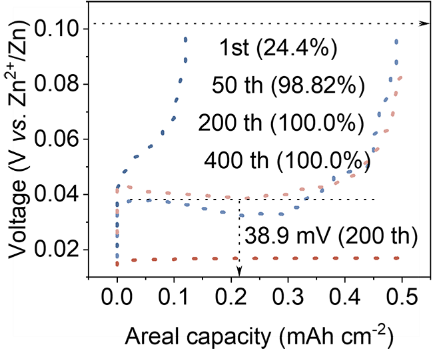


**Fig. S13** Voltage profiles of Zn//SS asymmetric cells at 0.5 mA cm^-2^/2.0 mAh cm^-2^ of HRP/CNF membrane


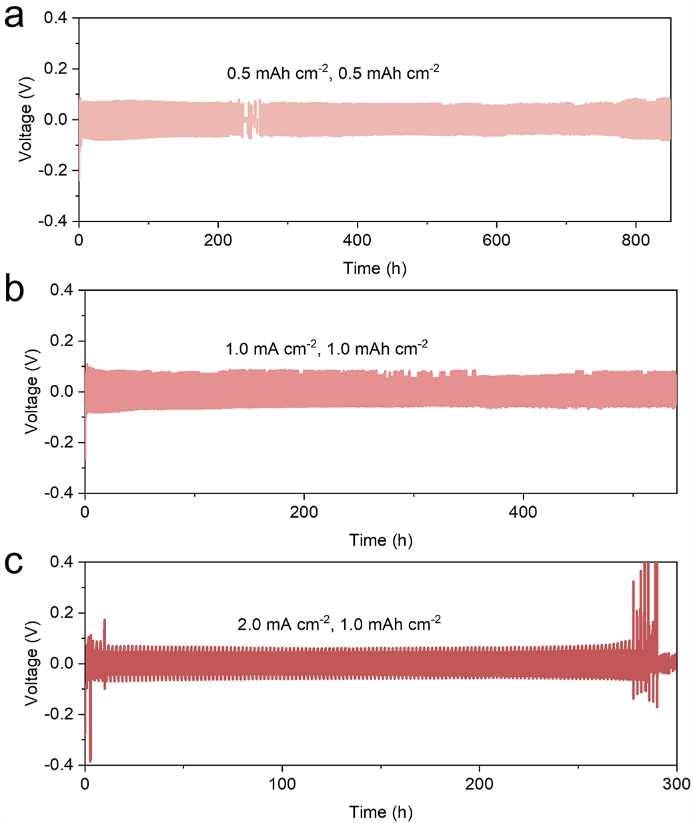


**Fig. S14** Potential-time curves within toilet paper/CNF membrane under different current densities of **a**) 0.2 mA cm^-2^, **b**) 1.0 mA cm^-2^, and **c**) 2.0 mA cm^-2^


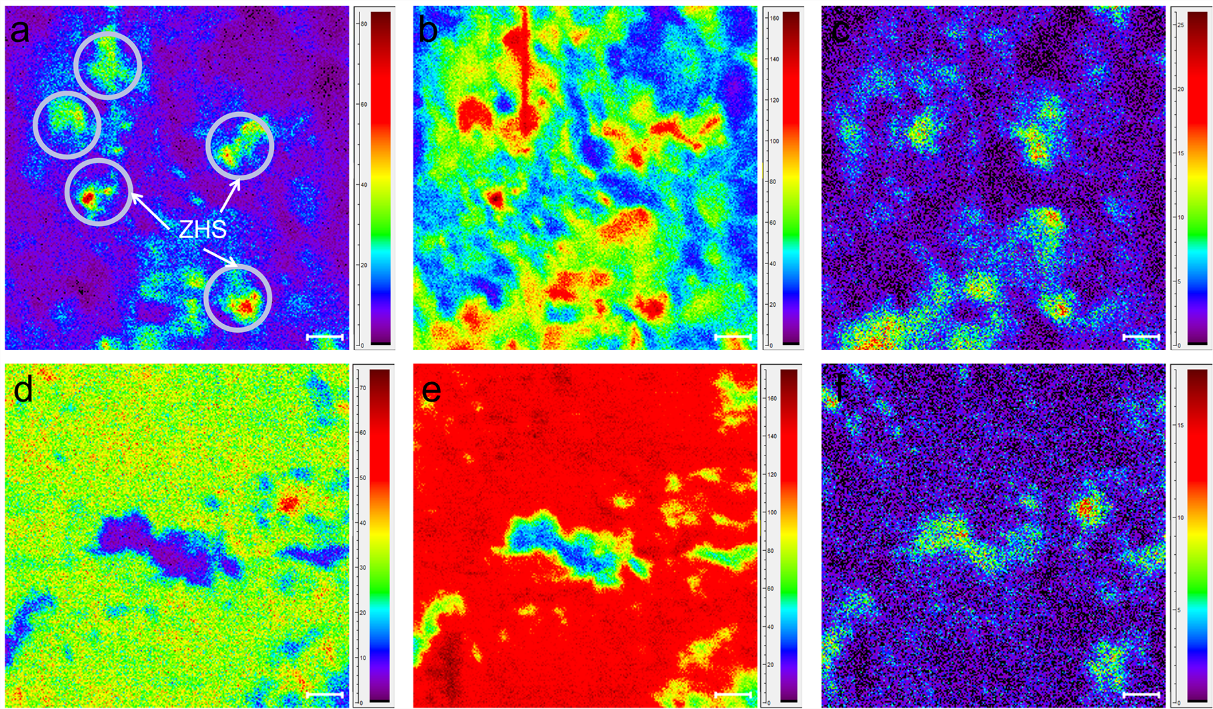


**Fig. S15** 2D TOF-SIMS images of anodes in S species **a**) GF, **d**) RP/CNF; O species **b**) GF, **e**) RP/CNF; C species **c**) GF, **f**) RP/CNF


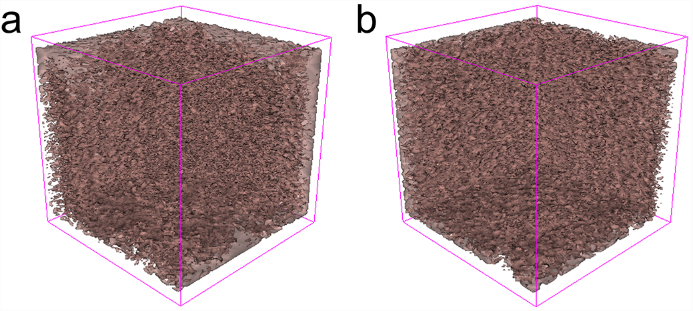


**Fig. S16** 3D TOF-SIMS results of zinc anode after plating with S species in **a**) GF and **b**) RP/CNF


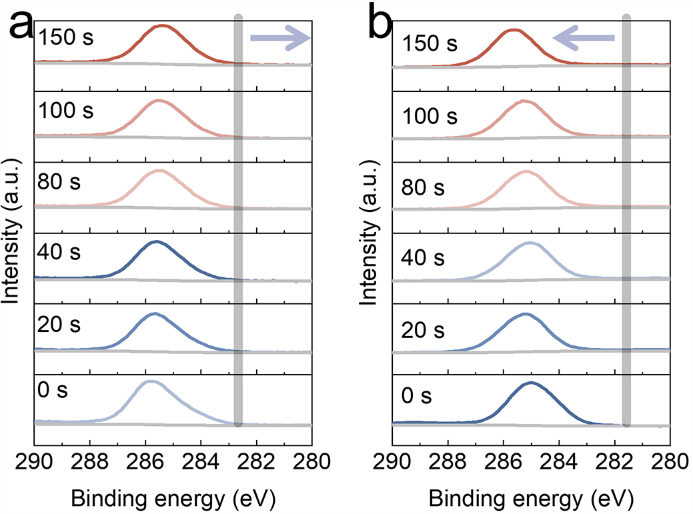


**Fig. S17** Depth profile of zinc anodes of C 1s in **a**) GF and **b**) RP/CNF membranes, respectively


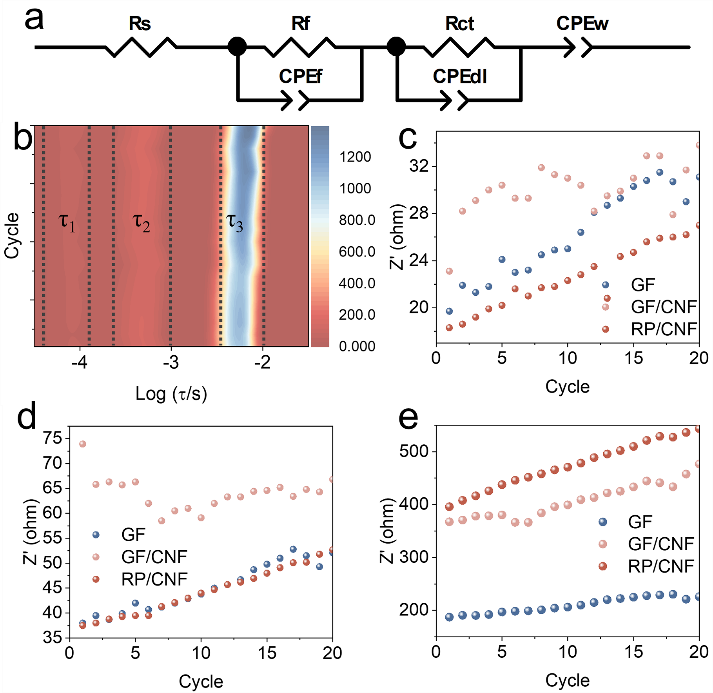


**Fig. S18 a**) Equivalent model for EIS simulation. **b**) Contour plot of DRT spectrum in GF/CNF membrane. **c**) R_sei_, **d**) R_ct_, **e**) R_diff_ values simulated from EIS under different plating times


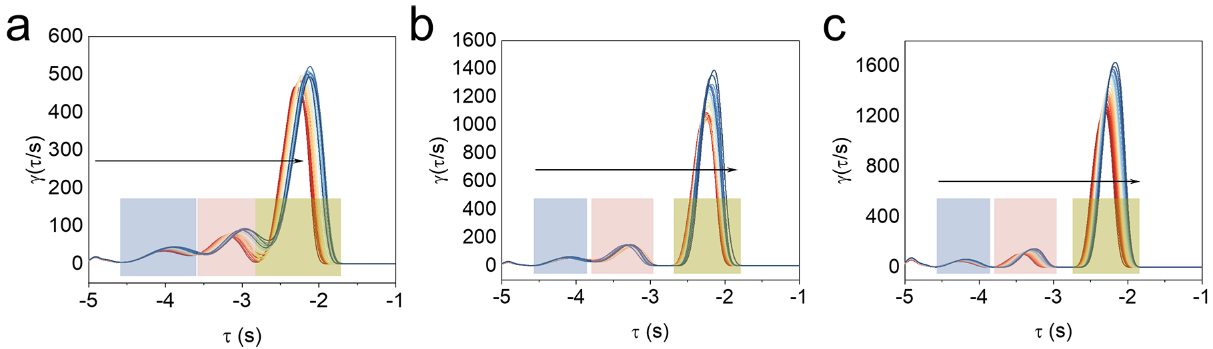


**Fig. S19** DRT spectra in different membranes: **a**) GF, **b**) GF/CNF, **c**) RP/CNF


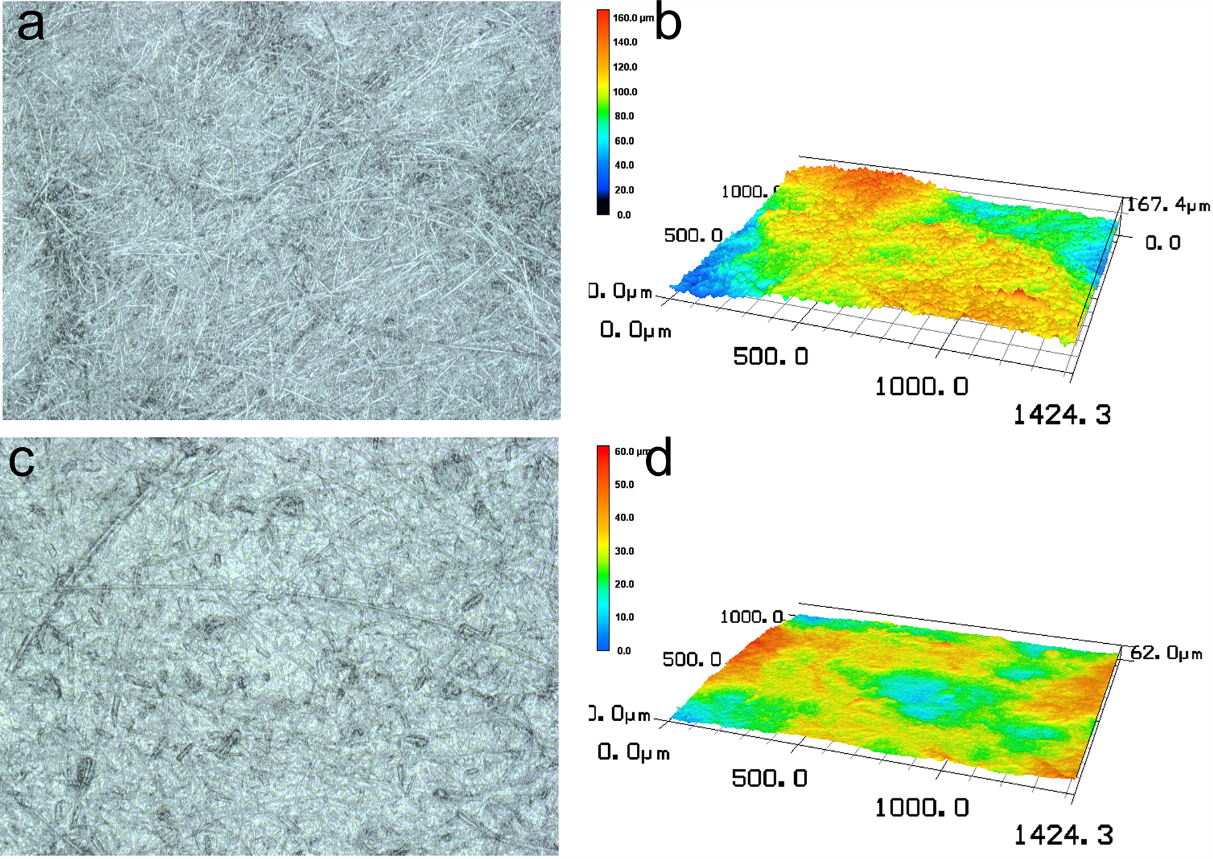


**Fig. S20** 3D laser scanning microscopy: **a, b**) GF, **c, d**) GF/CNF


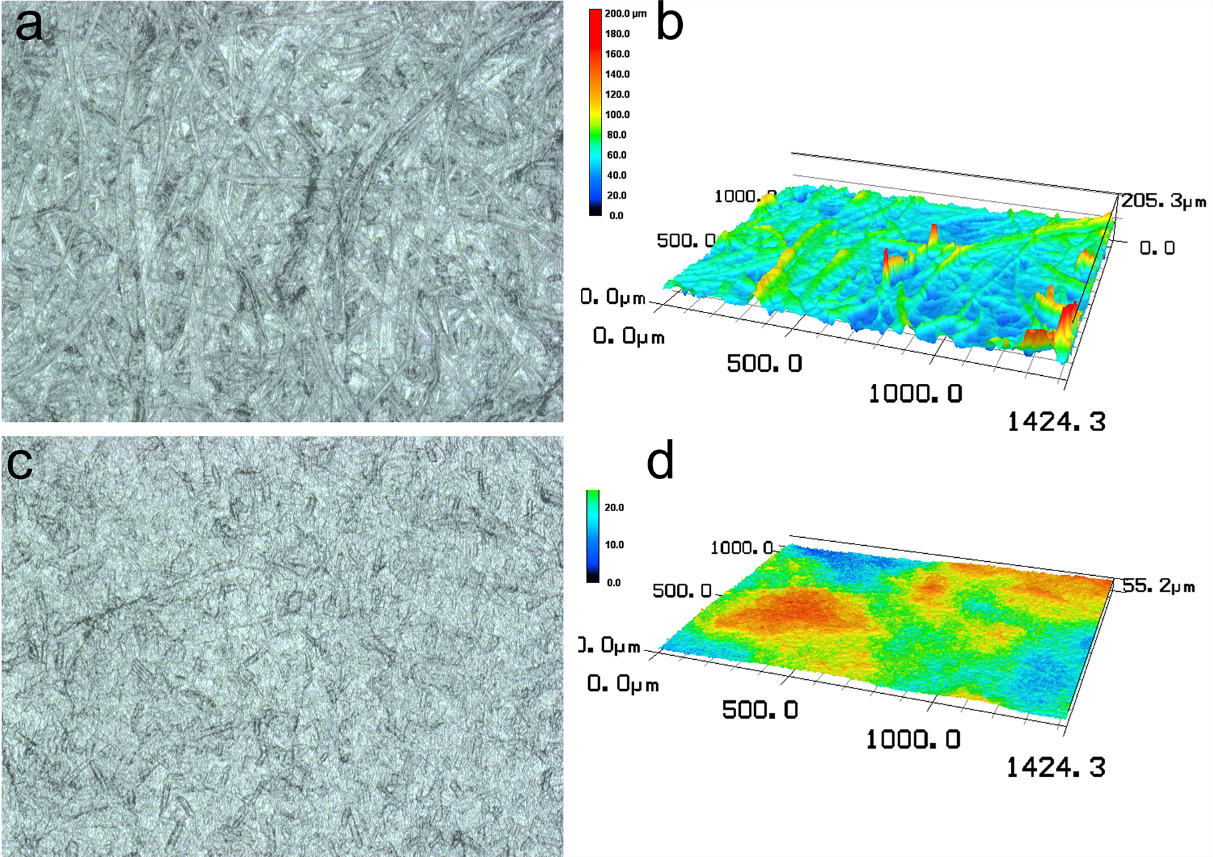


**Fig. S21** 3D laser scanning microscopy: **a, b**) RP, **c, d**) RP/CNF


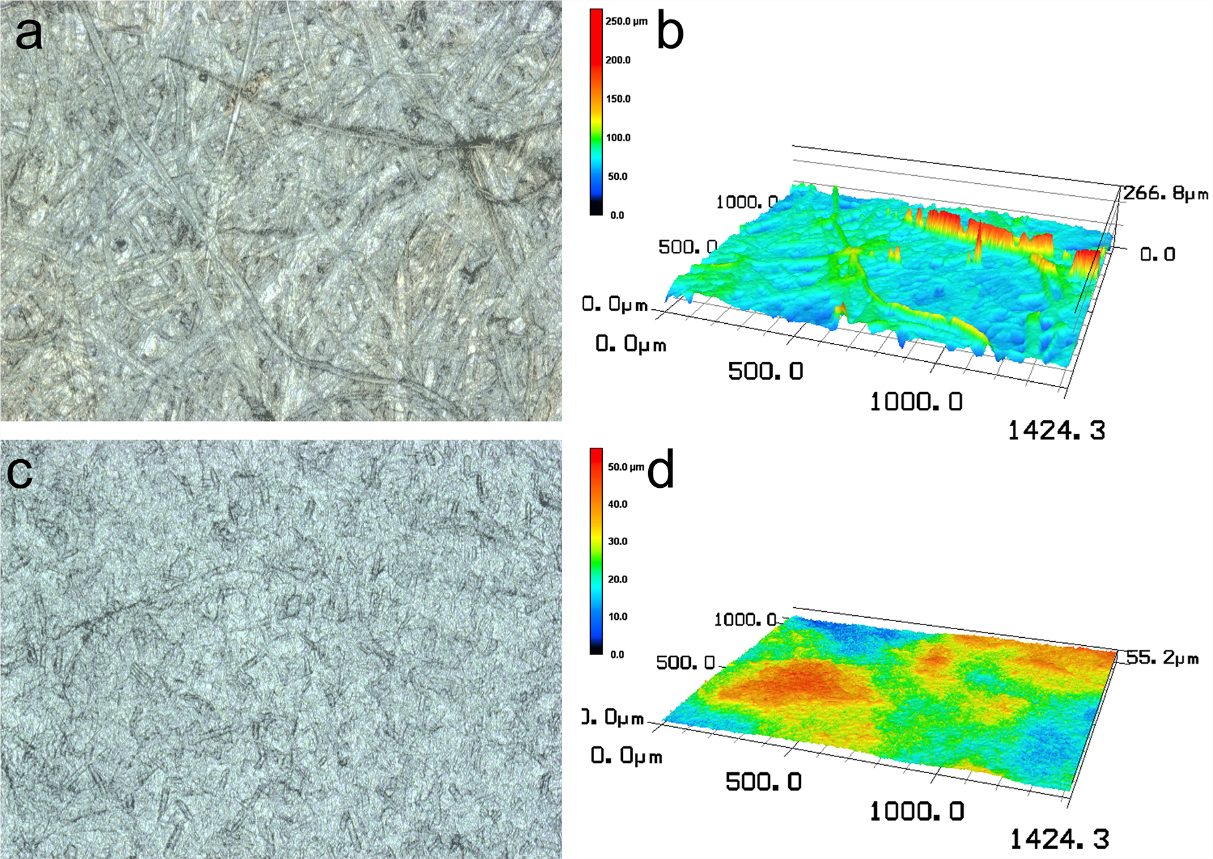


**Fig. S22** 3D laser scanning microscopy: **a, b**) HRP, **c, d**) HRP/CNF


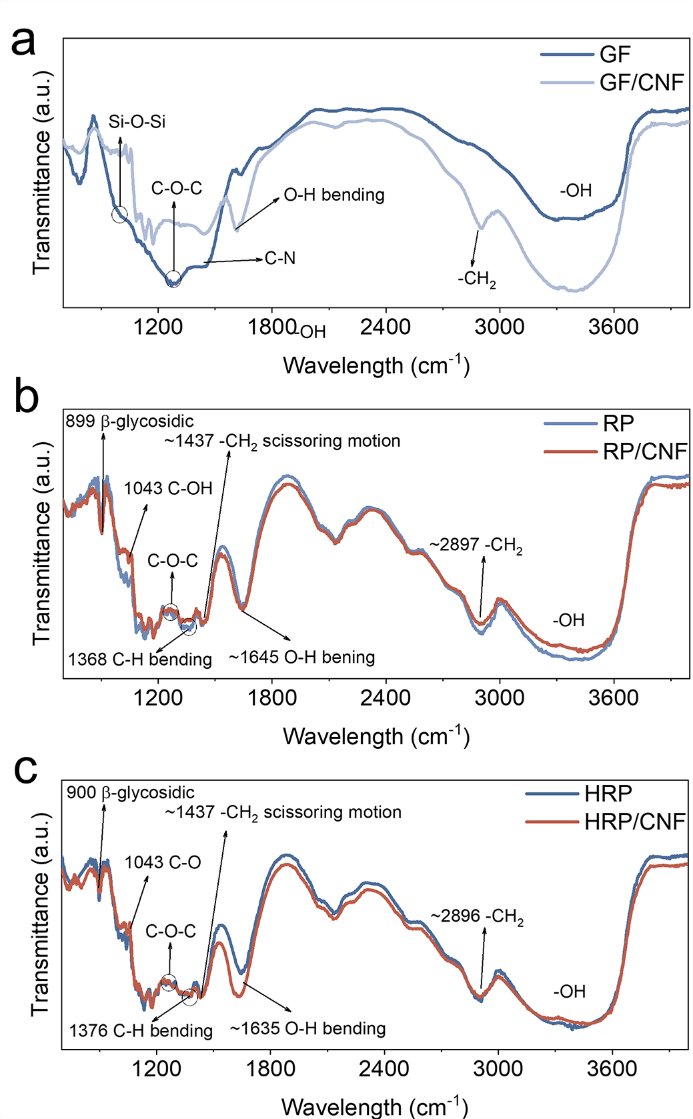


**Fig. S23** FTIR spectra in different membranes: **a**) GF, **b**) RP, and **c**) HRP


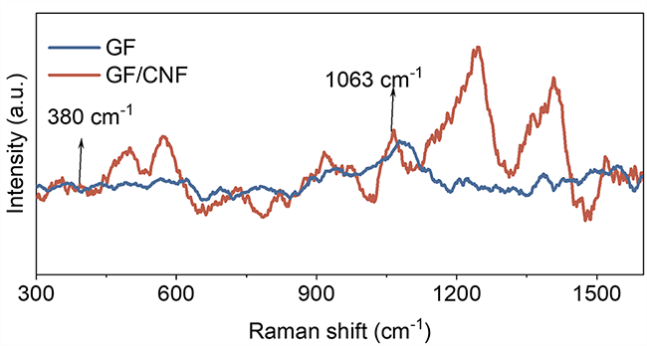


**Fig. S24** Raman spectra of GF and GF/Raman membrane


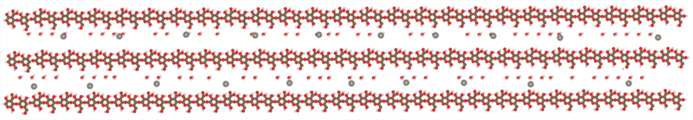


**Fig. S25** Poly-cellulose model with H_2_O and Zn^2+^ for MD simulation


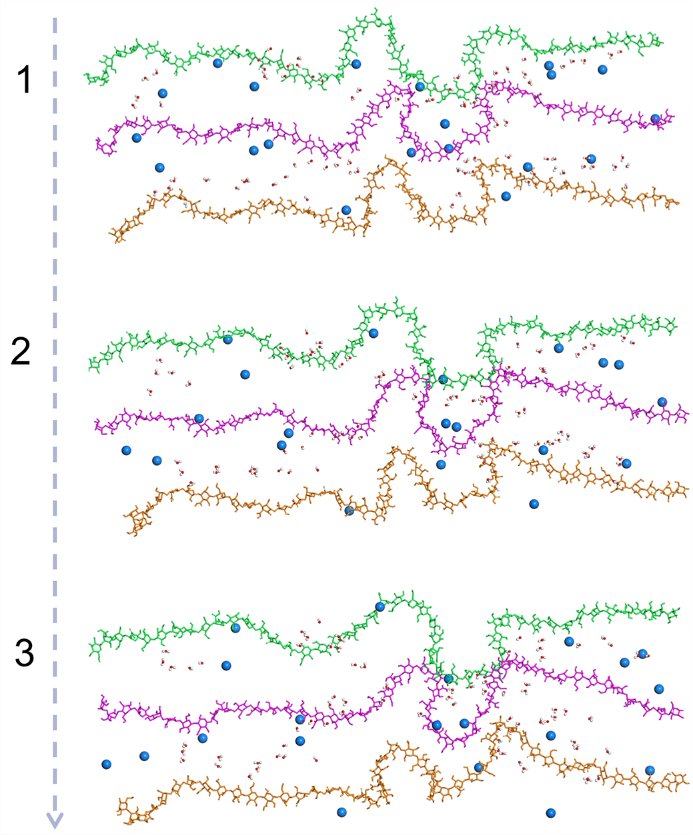


**Fig. S26** Energy barrier under diffusion path


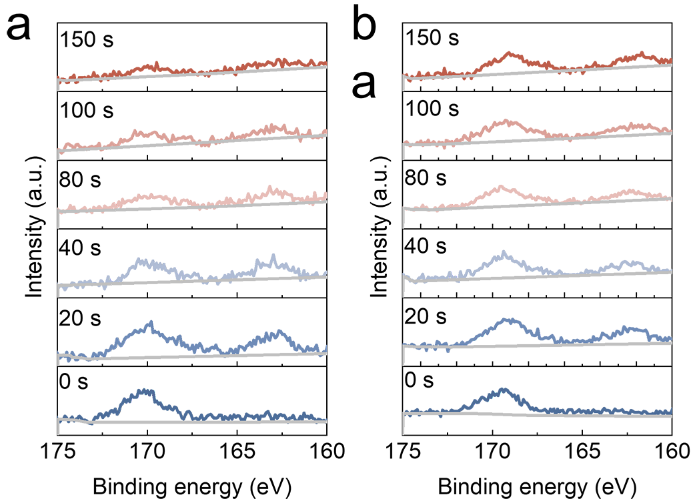


**Fig. S27** Depth profile of zinc anodes of S 2p in **a**) GF, and **b**) RP/CNF


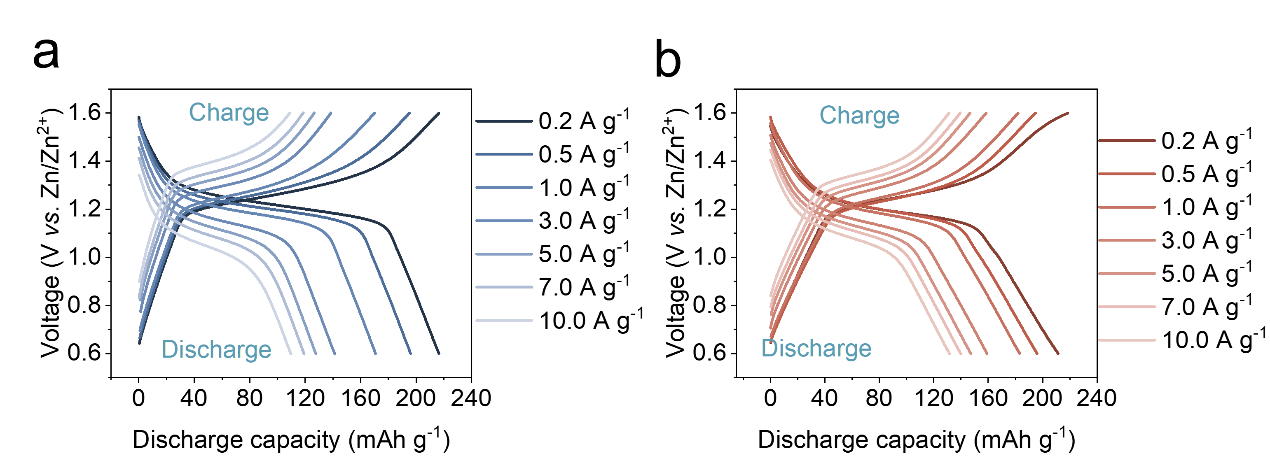


**Fig. S28** Charge and discharge curves in Zn||I_2_ batteries of **a**) RP and **b**) RP/CNF at different rates from 0.2 -10.0 A g^-1^


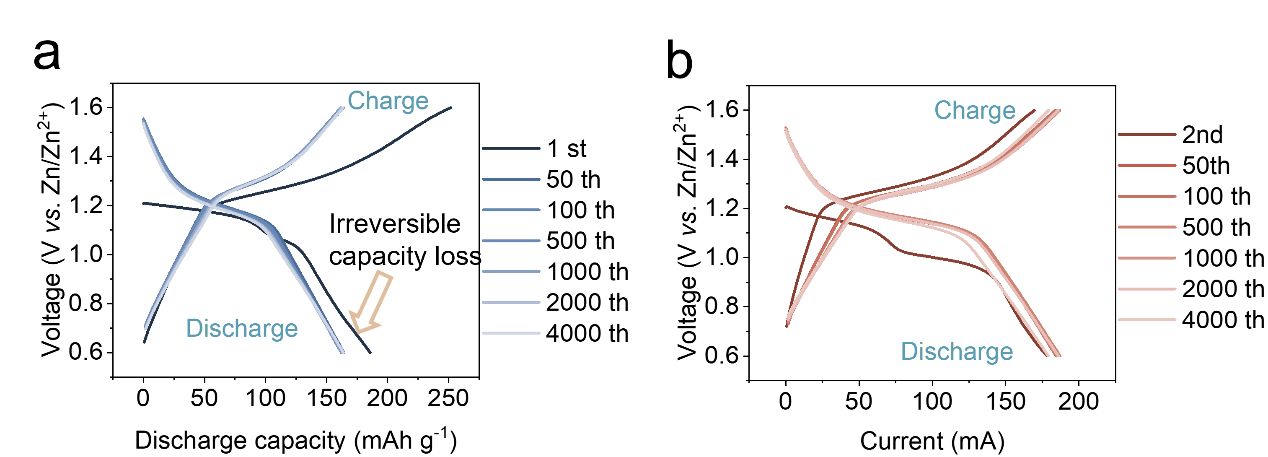


**Fig. S29** Charge and discharge curves in Zn||I_2_ batteries of **a**) GF and **b**) RP/CNF in long-term cycling

**Table S1** Comparison chart of the charge rate, capacity retention and corresponding cycle number of the batteries with other separators

| Separators | Battery Configuration | Charge Rate (A g^-1^) | Capacity Retention (mAh g^-1^) | Cycle Number | Refs. |
| --- | --- | --- | --- | --- | --- |
| PVA/CNF hydrogel | Zn\|\|MnO_2_ | 1.0 | 172.8 | 800 | [S2] |
| Chitosan/PIL Hydrogel | Zn\|\|NVO | 2.0 | < 150 | 2000 | [S3] |
| PVA/HPMC Hydrogel | Zn\|\|Zn-MnO | 2.0 | < 100 | 1000 | [S4] |
| Halloysite Nanotubes/Bacterial Cellulose | Zn\|\|Electrolytic MnO_2_ | 3C (~ 0.93) | 136.9 | 800 | [S5] |
| Gra-CeF_3_-GF | Zn\|\|V_2_O_5_ | 2.0 | ~ 100 | 2000 | [S6] |
| Halloysite Nanotubes/Cellulose | Zn\|\|V_2_O_5_ | 1.0 | 102 | 1000 | [S7] |
| ZnO-Halloysite Filter Paper | Zn‖MnO_2_ | 2.0 | < 120 | 1000 | [S8] |
| MOF-NS/PAN | Zn\|\|VSe_2_ | 1.0 | 120.1 | 1500 | [S9] |
| BC@UiO-66 | Zn\|\|MnO_2_ | 1.0 | 163.0 | 1000 | [S10] |
| GF-Bio-MOF-100 | Zn\|\|MnO_2_ | 0.5 | 141.1 | 1000 | [S11] |
| B/CNF | Zn\|\|VO_2_ | 1.0 | 151.2 | 500 | [S12] |
| BC-FP | Zn\|\|V_2_O_5_ | 1.0 | 90.0 | 2000 | [S13] |
| Dual-scale Asymmetric Cellulose | Zn\|\|I_2_ | 2.0 | 172.8 | 4000 | This work |

**Supplementary References**

1. S. Deng, Z. Tie, F. Yue, H. Cao, M. Yao et al., Rational design of ZnMn_2_O_4_ quantum dots in a carbon framework for durable aqueous zinc-ion batteries. Angew. Chem. Int. Ed. **61**(12), e202115877 (2022). <https://doi.org/10.1002/anie.202115877>
2. Q. Wang, J. Huang, L. Qi, M. Li, S. Wang et al., A bioinspired gradient hydrogel electrolyte network with optimized interfacial chemistry toward robust aqueous zinc-ion batteries. ACS Nano **19**(29), 26770–26781 (2025). <https://doi.org/10.1021/acsnano.5c06914>
3. M. Xu, F. Liu, L. Chen, Y. Lei, Z. Liu et al., Zwitterionic poly(ionic liquid) hydrogel electrolytes with high-speed ion conduction channels for dendrite-free, long-enduring zinc-ion batteries and flexible electronics. Energy Storage Mater. **80**, 104373 (2025). <https://doi.org/10.1016/j.ensm.2025.104373>
4. P.P. Puthiyaveetil, R.M. Kurian, N.S. Samudre, R. Balasubramanian, A. Torris et al., Self-healing hydrogel electrolyte enabled by dynamic polar covalent and noncovalent interactions for high-performance rechargeable zinc-metal batteries: a leap toward sustainable energy storage. Adv. Energy Mater. **16**(2), e02883 (2026). <https://doi.org/10.1002/aenm.202502883>
5. P. Xu, Y. Yu, B. Du, Y. Cao, D. Sun et al., High performance Janus separator based on microstructurally controllable halloysite nanotubes for zinc-ion batteries. J. Energy Storage **114**, 115820 (2025). <https://doi.org/10.1016/j.est.2025.115820>
6. Z. Zhao, Y. Zhang, H. Zhang, X. Shi, H. Zhao et al., Gradient structured separator enables stable aqueous zinc metal batteries. Nano Lett. **25**(18), 7483–7490 (2025). <https://doi.org/10.1021/acs.nanolett.5c01125>
7. M. Wang, Z. Dai, C. Yang, D. Xu, X. Zhang et al., Boosting de-solvation *via* halloysite nanotubes-cellulose composite separator for dendrite-free zinc anodes. Mater. Today Energy **46**, 101736 (2024). <https://doi.org/10.1016/j.mtener.2024.101736>
8. K. Yang, Y. Li, F. Zhang, B. Xue, X. Gu, A ZnO-halloysite coated composite separator capable of stabilizing zinc electrode interface for high-performance aqueous zinc-ion battery. Mater. Today Chem. **45**, 102651 (2025). <https://doi.org/10.1016/j.mtchem.2025.102651>
9. C.-Y. Liu, Y.-D. Wang, H. Liu, Q. Chen, X. Jiang et al., Channel engineering strategy of precisely modified MOF/nanofiber composite separator for advanced aqueous zinc ion batteries. Compos. Part B Eng. **272**, 111227 (2024). <https://doi.org/10.1016/j.compositesb.2024.111227>
10. T. Zhao, P. Xiao, S. Nie, J. Yu, S. Peng et al., Innovative bacterial cellulose and UiO-66 composites for superior zinc ion battery separator performance. Green Chem. **27**(31), 9541–9558 (2025). <https://doi.org/10.1039/d5gc01051a>
11. R. Li, B. Yan, Z. Chen, Z. He, J. Yang, A multifunctional separator decorated by anionic metal-organic framework toward ultrastable zinc anodes. J. Energy Chem. **105**, 860–868 (2025). <https://doi.org/10.1016/j.jechem.2025.01.043>
12. J. Cao, X. Rao, S. Qian, D. Zhang, Y. Jin et al., Dynamic Zn^2+^-coordinating oxygen sites and electric field modulation in boron-integrated cellulose nanofiber separators for stable zinc-ion batteries. Adv. Energy Mater. **15**(47), e03368 (2025). <https://doi.org/10.1002/aenm.202503368>
13. Z. Liu, Q. Liu, H. Mo, Y. Duan, J. Zhang et al., Gradient cellulose-based separator with dual nano-confinement and ion-guiding functions for dendrite-free zinc-ion batteries. Chem. Eng. J. **523**, 168579 (2025). <https://doi.org/10.1016/j.cej.2025.168579>
